# Young People’s Mental Health Changes, Risk, and Resilience During the COVID-19 Pandemic

**DOI:** 10.1001/jamanetworkopen.2023.35016

**Published:** 2023-09-21

**Authors:** Jesus Montero-Marin, Verena Hinze, Karen Mansfield, Yasmijn Slaghekke, Sarah-Jayne Blakemore, Sarah Byford, Tim Dalgleish, Mark T. Greenberg, Russell M. Viner, Obioha C. Ukoumunne, Tamsin Ford, Willem Kuyken, and the MYRIAD Team

**Affiliations:** 1 Department of Psychiatry, Warneford Hospital, University of Oxford, Oxford, England, United Kingdom; 2 Teaching, Research & Innovation Unit, Parc Sanitari Sant Joan de Déu, Sant Boi de Llobregat, Spain; 3 Consortium for Biomedical Research in Epidemiology and Public Health (CIBER Epidemiology and Public Health-CIBERESP), Madrid, Spain; 4 Department of Psychology, University of Cambridge, Cambridge, England, United Kingdom; 5 Institute of Psychiatry, Psychology & Neuroscience, King’s Health Economics, King’s College London, London, England, United Kingdom; 6 Medical Research Council Cognition and Brain Sciences Unit, University of Cambridge, Cambridge, England, United Kingdom; 7 Edna Bennett Pierce Prevention Research Center, College of Health and Human Development, Pennsylvania State University, State College, Pennsylvania; 8 Population, Policy and Practice Research and Teaching Department, University College London Great Ormond Street Institute of Child Health, London, England, United Kingdom; 9 National Institute for Health and Care Research Applied Research Collaboration (PenARC) South West Peninsula, Department of Health and Community Sciences, Faculty of Health and Life Sciences, University of Exeter, Exeter, England, United Kingdom; 10 Department of Psychiatry, School of Clinical Medicine, University of Cambridge, Cambridge, England, United Kingdom

## Abstract

**Importance:**

As young people’s mental health difficulties increase, understanding risk and resilience factors under challenging circumstances becomes critical.

**Objective:**

To explore the outcomes of the COVID-19 pandemic on secondary school students’ mental health difficulties, as well as the associations with individual, family, friendship, and school characteristics.

**Design, Setting, and Participants:**

For this cohort study, follow-up data from the My Resilience in Adolescence (MYRIAD) cluster randomized clinical trial were collected across 2 representative UK cohorts. Mainstream UK secondary schools with a strategy and structure to deliver social-emotional learning, with an appointed head teacher, and that were not rated “inadequate” in their latest official inspection were recruited. A total of 5663 schools were approached, 532 showed interest, and 84 consented. Cohort 1 included 12 schools and 864 students, and cohort 2 included 72 schools and 6386 students. COVID-19 was declared a pandemic after cohort 1 had completed all assessments (September 2018 to January 2020), but cohort 2 had not (September 2019 to June 2021).

**Exposures:**

Cohort 2 was exposed to the COVID-19 pandemic, including 3 national lockdowns. Associations of individual, family, friendship, and school characteristics with students’ mental health were explored.

**Main Outcomes and Measures:**

Changes in students’ risk for depression (Center for Epidemiological Studies-Depression scale); social, emotional, and behavioral difficulties (Strengths and Difficulties Questionnaire); and mental well-being (Warwick-Edinburgh Mental Well-Being Scale).

**Results:**

Of the 7250 participants included, the mean (SD) age was 13.7 (0.6) years, 3947 (55.4%) identified as female, and 5378 (73.1%) self-reported their race as White. Twelve schools and 769 of the 864 students (89.0%) in cohort 1 and 54 schools and 2958 of the 6386 students (46.3%) in cohort 2 provided data and were analyzed. Mental health difficulties increased in both cohorts but to a greater extent among students exposed to the pandemic, including for risk of depression (adjusted mean difference [AMD], 1.91; 95% CI, 1.07-2.76); social, emotional, and behavioral difficulties (AMD, 0.76; 95% CI, 0.33-1.18); and mental well-being (AMD, −2.08; 95% CI, −2.80 to −1.36). Positive school climate, high home connectedness, and having a friend during lockdown were protective factors during the pandemic. Female gender and initial low risk for mental health difficulties were associated with greater mental health deteriorations. Partial school attendance during lockdown was associated with better adjustment than no attendance when returning to school.

**Conclusions and Relevance:**

This cohort study of secondary school students demonstrated that to promote mental health and adjustment, policy interventions should foster home connectedness, peer friendship, and school climate; avoid full school closures; and consider individual differences.

## Key Points

**Question:**

Did secondary school students’ mental health difficulties and mental well-being change during the COVID-19 pandemic, and what factors are associated with risk and resilience?

**Findings:**

In this cohort study of 7250 UK students not exposed (12 schools; n = 864 students) and exposed (72 schools; n = 6386 students) to the pandemic during assessment, greater risk for depression; social, emotional, and behavioral difficulties; and deteriorations in mental well-being were associated with the pandemic. Risk and resilience were associated with individual, home, friendship, and school characteristics.

**Meaning:**

The COVID-19 pandemic was associated with worsened outcomes in mental health for secondary school students, and their risk and resilience needs to be conceptualized at individual and social levels.

## Teaser

This cohort study explores the outcomes of the COVID-19 pandemic on secondary school students’ mental health difficulties, as well the associations with individual, family, friendship, and school characteristics.

## Introduction

There is growing evidence that the prevalence of mental health conditions in young people, particularly girls,^1^ is increasing in the UK.^2^ From the start of 2020, young people’s lives were disrupted by restrictions resulting from the COVID-19 pandemic.^3^ Observational studies have reported mixed findings but have mostly suggested that young people’s mental health deteriorated.^4,5^ This was possibly related to disruptions to school and social interactions, COVID-19–related anxieties, family illness, grief from possible loss of relatives, economic effects, ongoing uncertainty, and reduced access to mental health services and other support.^6,7,8,9,10^ However, the lack of a suitable comparison group means that the specific role of COVID-19 and underpinning risk and resilience factors remains unclear.

To advise immediate and future policy decisions, better understanding is needed on what factors affect young people’s mental health, particularly during challenging circumstances.^11^ Informed by theoretical models and previous research,^12,13,14^ we explored the associations of individual, home, friendship, and school (eg, school community, operational features of the school, the broader school context) factors with mental health difficulties and mental well-being for secondary school students (hereafter, students) before and during the pandemic. Students in the UK aged 11 to 13 years were recruited in 2 cohorts (enrolled in 2016 and 2017, respectively). Participants were followed up over a 3-year period, which included the COVID-19 pandemic for the second cohort only.^15^ This created a natural experiment ideally suited to evaluate the association of the pandemic with students’ mental health difficulties and mental well-being.^16^

We aimed to disentangle known difficulties in students’ mental health and mental well-being from changes specific to the COVID-19 pandemic. We also secondarily explored which individual, home, friendship, and school factors were associated with changes in students’ mental health difficulties and mental well-being during the pandemic and with adjustment to lockdown and return to school.

## Methods

### Study Design and Participants

**Figure 1.**
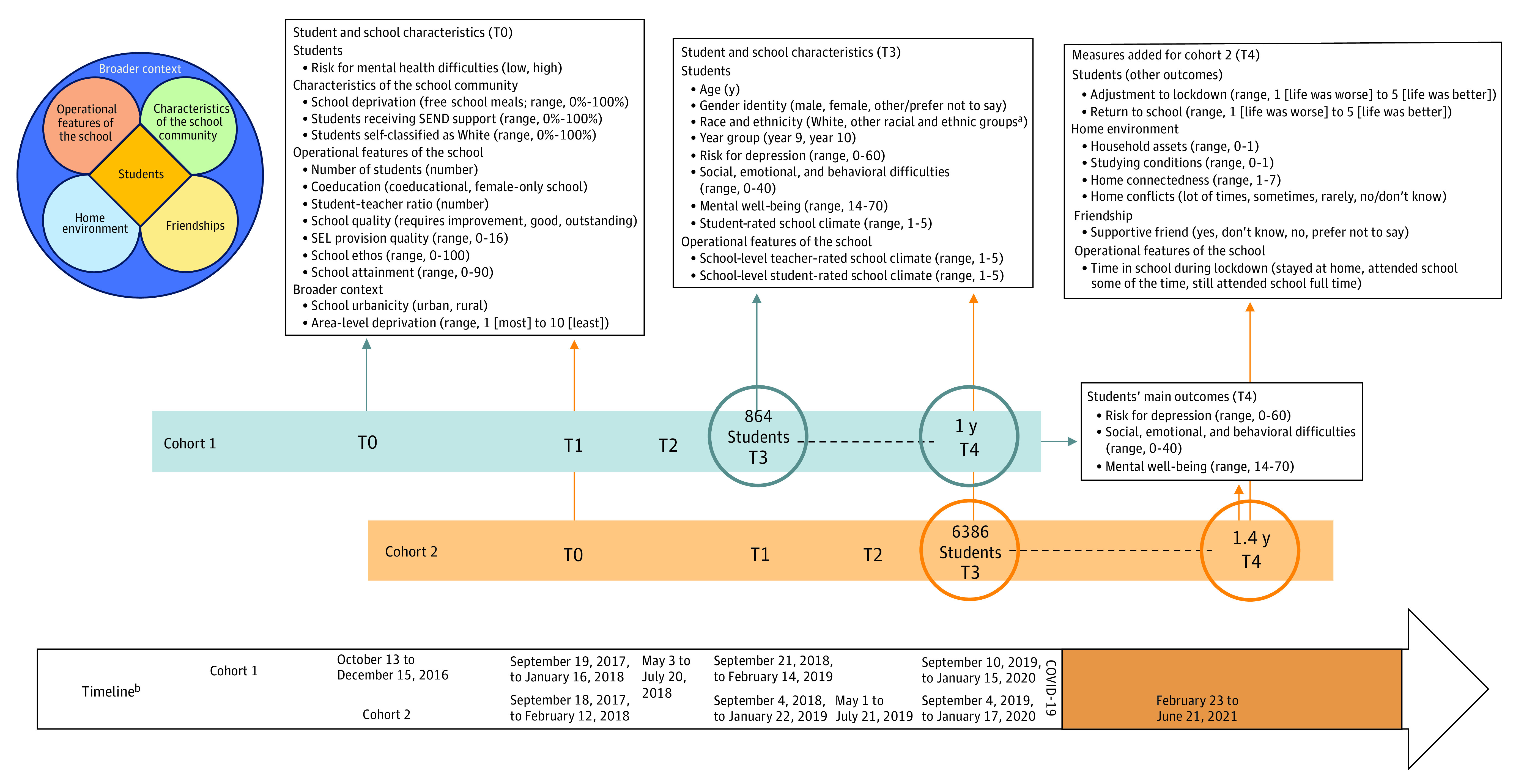
Study Design, Measures, and Data Collection Student and school characteristics (characteristics of the school community, operational features of the school, and broader context), home environment, and friendships may influence students’ mental health, mental well-being, and adjustment to lockdown and return to school directly or indirectly through different mechanisms.^12^ SEL indicates social-emotional learning; SEND, special educational needs or disabilities. ^a^The other racial and ethnic groups category includes Arab, Asian, Black/African/Caribbean, mixed/multiple ethnic groups, and other ethnic groups. This category was grouped together owing to small sample sizes. ^b^The time point 3 (T3) to time point 4 (T4) window for cohort 1 was a mean (SD) of 364.6 (50.9) days and for cohort 2 was 523.9 (47.4) days. The UK government announced on March 23, 2020, that residents must stay at home and some businesses had to close. This lockdown was gradually eased until July 4, 2020, when most businesses were allowed to open. From late July 2020 onward, a series of limited and local restrictions were put in place. From November 5, 2020, the UK went into a second, 4-week lockdown, which differed from the first in that schools and universities remained open, and from December 2020 onward returned to a system of local restrictions. From December 26, 2020, the UK went into a third lockdown with a gradual end: in March 2021, schools reopened; in April 2021, individuals in groups of up to 6 were allowed to meet again outdoors, and outdoor pubs, restaurants, and nonessential shops reopened in the UK; in mid-May 2021, outdoor social contact became unregulated again, individuals in groups of up to 6 were allowed to meet indoors, and restaurants and hotels could reopen. See eFigure 1 in Supplement 1 for a more detailed description of the pandemic timeline and lockdown restrictions at the T4 measurement wave for cohort 2.

Figure 1 shows the study design. COVID-19 was declared a pandemic after cohort 1 (n = 12 schools) had completed the data collection for all follow-up time points (time point 3 [T3], autumn 2018; time point 4 [T4], autumn 2019). For cohort 2 (n = 72 schools), COVID-19 was declared a pandemic between the 1- and 2-year follow-up time points (T3, autumn 2019; T4, winter 2020/spring 2021), creating optimal conditions for a natural experiment. We collected T4 measures for cohort 2 during the third UK lockdown, when adults were encouraged to work from home, social activities were restricted, and schools were mostly open, although classes and sometimes entire year groups were asked to work remotely when high numbers of students tested positive for COVID-19. Schools reopened after the first lockdown in the summer and autumn of 2020. Hence, all students in cohort 2 had the experience of returning to school after a national lockdown prior to T4. The pandemic timeline and lockdown restrictions are described in eFigure 1 in Supplement 1.

Data were collected as part of the My Resilience in Adolescence (MYRIAD) trial. The MYRIAD trial is a 2-arm cluster randomized clinical trial that examined whether school-based mindfulness training improved students’ mental health.^15,16^ Overall, 8376 students in 84 UK secondary schools were recruited in the academic years 2016 to 2017 (cohort 1) and 2017 to 2018 (cohort 2).^17^ Schools were eligible if they were mainstream UK secondary schools with a strategy and structure in place to deliver social-emotional learning (SEL), an appointed head teacher, and if they had not been rated “inadequate” in their latest official school inspection.

The MYRIAD trial and present study were approved by the University of Oxford Medical Sciences Division Ethics Committee (R45358/RE001 and R45358/RE023, respectively) and overseen by a data monitoring and ethics committee and the MYRIAD trial steering committee. Written informed consent/assent was collected from schools, parents, and students. We followed the Strengthening the Reporting of Observational Studies in Epidemiology (STROBE) reporting guideline for cohort studies.^18^

### Measures

Figure 1 shows the timeline for all measures, their conceptualization, and metrics. Further details on the measures can be found in the eMethods in Supplement 1.

#### Main Outcomes

Self-report measures were used to assess students’ risk for depression (Center for Epidemiological Studies-Depression scale)^19^; social, emotional, and behavioral difficulties (Strengths and Difficulties Questionnaire)^20^; and mental well-being (Warwick-Edinburgh Mental Well-Being Scale).^21,22^

#### Other Outcomes

Adjustment to lockdown and return to school were assessed in cohort 2 only using 2 newly developed items asking about the extent to which students perceived that their lives were affected by (1) the lockdowns and (2) return to school after the lockdowns. Questions were answered on a 5-point Likert scale from 1 (“life was much worse”) to 5 (“life was much better”).

#### Explanatory Factors

We assessed theory-driven individual, home, friendship, and school factors that might be associated with changes in students’ mental health over time.^12,13,14^ Student-level individual factors included age, gender identity, self-classified race and ethnicity, year group, student-rated school climate,^23^ and initial risk for mental health difficulties.^24^ Student-level factors related to the home environment included household assets,^25^ studying conditions, home connectedness,^26^ and home conflicts. Student-level factors related to the social context during lockdown included friendships. School-level factors included the school community (percentage of students eligible for free school meals [ie, school deprivation], receiving support for special educational needs or disabilities, and students who were White [Ford et al^12^ suggest a potential association of this variable at the school level with mental health outcomes at the student level]),^24^ operational school features (number of students, student-to-teacher ratio, coeducation, school quality,^27^ SEL provision quality,^17^ school SEL ethos,^24^ school attainment,^28^ school-level teacher-rated and student-rated school climate,^23^ and time in school during the third lockdown), and the broader school’s context (urbanicity and area-level deprivation).^29^

### Statistical Analysis

#### Temporal Trends and Cohort Differences

We described students’ mental health difficulties and mental well-being over time separately for both cohorts. To reveal temporal trends, we summarized overall mean (SD) scores for risk for depression; social, emotional, and behavioral difficulties; and mental well-being at each time point and calculated standardized effect sizes using Hedges *g*.^30^ We explored missing data, carried out complete-case analyses (proportion of missingness >40%),^31^ and evaluated how it affected statistical power. To assess whether changes in students’ mental health difficulties and mental well-being differed by cohort, we fitted 3-level mixed-effects linear regressions via maximum likelihood estimation (allowing for dependencies between repeated observations nested within participants and within schools). First, we estimated the time effect (ie, T3-T4 change) for cohort 1 and cohort 2 separately, then we evaluated the time-by-cohort interaction.

#### Associations With Changes in Students’ Mental Health and Well-Being

These explorations were undertaken for cohorts 1 and 2 separately. Both cohorts included student- and school-level factors (student-level characteristics related to the home environment and friendships were explored in cohort 2 only). We fitted 3-level mixed-effects linear regressions, using maximum likelihood estimation in 2 steps. First, we examined 1 factor at a time for each outcome. If the inclusion of the time-by-factor term provided both a significant coefficient for the interaction and a better model fit (likelihood ratio test), compared with models with only the main effects (time and the factor), this evidenced that the factor-outcome association changed between T3 and T4. Second, to evaluate unique associations, we fitted multivariable models, entering those factors (and interactions, if relevant) that provided statistically significant *P* values in the first step.

#### Associations With Students’ Adjustment to Lockdown and Return to School

For cohort 2, we explored whether characteristics of the individual, home environment, friendship, school community, operational features of the school, and the broader context were associated with students’ adjustment during lockdown and when returning to school at T4. First, we examined the associations between each factor and students’ adjustment in univariable models. We allowed for associations between observations nested within schools, using 2-level mixed-effects linear regressions. Second, we estimated unique associations by fitting multivariable models, with only those factors that had statistically significant *P* values in the univariable analyses.

All models were adjusted for design variables (country, school size, and coeducation)^15^ and time (days) between T3 and T4. Owing to the lack of intervention effects in the trial,^17^ we analyzed both trial arms together but adjusted for allocation. We used 2-sided tests with a significance level of *P* < .05 and controlled for the false discovery rate in all multivariable models.^32^ Analyses were performed using Stata, version 17.0 (StataCorp).

## Results

### Participant Demographics

Of the 7250 participants included, the mean (SD) age was 13.7 (0.6) years, 3947 (55.4%) identified as female, and 5378 (73.1%) self-reported their race as White. Table 1 summarizes the study sample at T3 (cohort 1: 864 students across 12 schools; cohort 2: 6386 students across 72 schools). Students in cohort 1 initially had lower mental health difficulties and higher mental well-being than students in cohort 2. Twelve schools and 769 students (89.0%) in cohort 1 (prepandemic) and 54 schools and 2958 students (46.3%) in cohort 2 (midpandemic) were retained until T4 and provided data on at least 1 outcome. Compared with students lost to follow-up, those retained reported slightly lower mental health difficulties and higher mental well-being at T3, and this was more pronounced for students in cohort 1 vs cohort 2 (eTable 1 in Supplement 1, which also describes missing data and how this affected statistical power). Both cohorts were comparable and representative of the UK general population in terms of school and student characteristics (eTable 2 in Supplement 1). eFigure 1 in Supplement 1 shows the number of students in cohort 2 responding at T4 in the context of the pandemic restrictions. eTable 3 in Supplement 1 summarizes students’ adjustment, home environment, and friendships during lockdown in cohort 2, as measured at T4. eFigure 2 in Supplement 1 shows student transitions from T3 to T4 by cohort, using validated cutoffs for the main outcomes. Results in the following sections were adjusted for multiple testing.

**Table 1.** Characteristics of Participating Students and Schools by Cohort^a^

Student characteristics	No. (%)	
	Cohort 1 (n = 864)	Cohort 2 (n = 6386)
Age, mean (SD), y	13.6 (0.6)	13.7 (0.6)
Gender		
Female	477 (55.3)	3470 (55.4)
Male	367 (42.5)	2671 (42.7)
Other/prefer not to say	19 (2.2)	116 (1.9)
Race and ethnicity		
White	708 (81.9)	4670 (73.1)
Other racial and ethnic groups^b^	156 (18.1)	1716 (26.9)
Year group^c^		
Year 9	508 (58.8)	3608 (56.5)
Year 10	356 (41.2)	2778 (43.5)
Student-rated school climate (range, 1-5), mean (SD)	3.1 (0.6)	3.1 (0.7)
Risk for depression (CES-D scale; range, 1-60), mean (SD)	15.9 (11.7)	17.0 (11.9)
Social, emotional, and behavioral difficulties (SDQ; range, 0-40), mean (SD)	12.5 (6.7)	13.2 (6.8)
Mental well-being (WEMWBS; range, 14-70), mean (SD)	48.0 (9.7)	47.5 (9.8)
Risk for mental health difficulties		
High	216 (25.0)	1647 (25.8)
Low	648 (75.0)	4739 (74.2)
**School characteristics**	**Cohort 1 (n = 12)**	**Cohort 2 (n = 72)**
School community		
Students eligible for free school meals, mean (SD), %	9.8 (8.1)	12.9 (9.6)
Students receiving SEND support, mean (SD), %	8.4 (3.8)	10.4 (5.8)
Students who are White, mean (SD), %^d^	83.5 (11.2)	68.6 (29.1)
School size (No. of students), mean (SD)	1144.2 (300.3)	991.2 (340.6)
Coeducation		
Coeducational school	11 (91.7)	62 (86.1)
Female only	1 (8.3)	10 (13.9)
Student-to-teacher ratio, mean (SD)	15.5 (1.3)	15.9 (1.9)
School quality (OFSTED)		
Outstanding	3 (25.0)	13 (18.1)
Good	7 (58.3)	39 (54.2)
Requires improvement	2 (16.7)	9 (12.5)
SEL quality rating (range, 1-16), mean (SD)	10.2 (3.8)	12.3 (2.2)
SEL ethos (range, 1-100), mean (SD)	47.7 (13.5)	50.3 (9.4)
School attainment (range, 9-90), mean (SD)	46.3 (5.8)	46.0 (15.7)
Teacher-rated school climate (range, 1-5), mean (SD)	3.8 (0.3)	3.9 (0.4)
Student-rated school climate (range, 1-5), mean (SD)	3.1 (0.2)	3.1 (0.2)
Broader school context, urbanicity		
Urban	10 (83.3)	61 (84.7)
Rural	2 (16.7)	11 (15.3)
Area-level deprivation (IMD; range, 1-10), mean (SD)	6.3 (2.9)	5.7 (2.7)

Abbreviations: CES-D, Center for Epidemiological Studies for Depression; IMD, Indices of Multiple Deprivation; OFSTED, Office for Standards in Education; SDQ, Strengths and Difficulties Questionnaire; SEL, social-emotional learning; SEND, special educational needs or disabilities; WEMWBS, Warwick-Edinburgh Mental Well-Being Scale.

^a^ In cohort 1, 863 students provided data on gender, 862 provided data on the CES-D, 860 provided data on the SDQ, and 843 provided data on school climate. In cohort 2, 6257 students provided data on gender, 6376 provided data on the CES-D, 6365 provided data on the SDQ, 6380 provided data on the WEMWBS, and 6243 provided data on school climate. In cohort 2, 66 schools provided data on percentage of students receiving SEND support, 63 provided data on percentage of students who are White, 61 provided data on school quality, 62 provided data on school attainment, and 70 provided data on teacher-rated school climate.

^b^ The other racial and ethnic groups category includes Arab, Asian, Black/African/Caribbean, mixed/multiple ethnic groups, and other ethnic groups. This category was grouped together owing to small sample sizes.

^c^ School year groups correspond across the home nations as follows: England, 9 and 10; Northern Ireland, 10 and 11; and Scotland, S2 and S3.

^d^ Ford et al^12^ suggest a potential association of this variable at the school level with mental health outcomes at the student level.

### Temporal Trends and Cohort Differences

**Figure 2.**
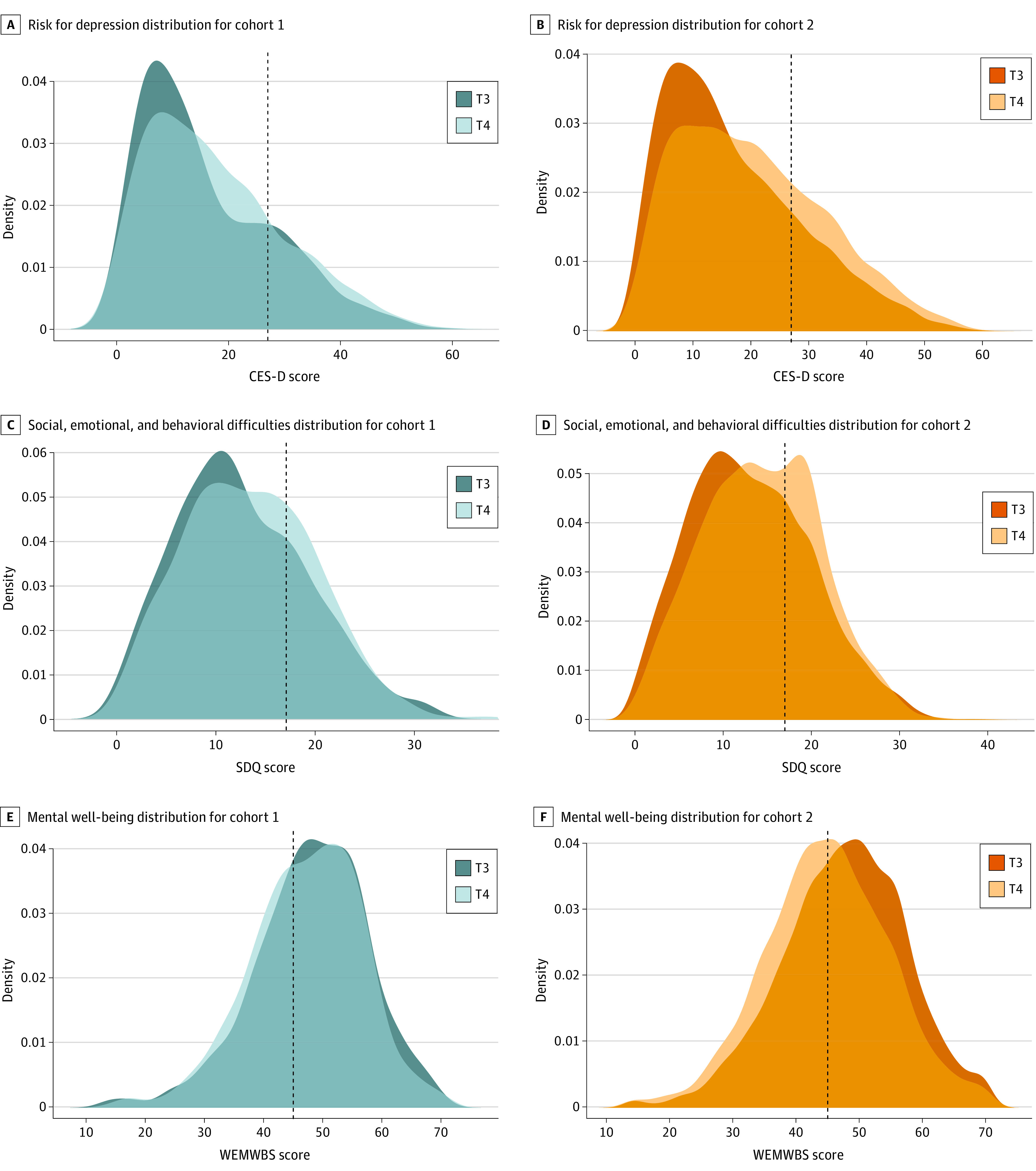
Outcomes by Cohort and Time Point A and B, Risk for depression was assessed using the Center for Epidemiological Studies for Depression scale (CES-D; range, 0-60; cutoff for caseness [dotted line], >27 points). C and D, Social, emotional, and behavioral difficulties were assessed using the Strengths and Difficulties Questionnaire (SDQ; range, 0-40; cutoff for high social, emotional, and behavioral difficulties [dotted line], >17 points). E and F, Mental well-being was assessed using the Warwick-Edinburgh Mental Well-Being Scale (WEMWBS; range, 14-70; cutoff for possible mental health difficulties [dotted line], <45 points).^19,20,21,22^ Time point 3 (T3) for cohort 1 was from September 21, 2018, to February 14, 2019, and for cohort 2 was September 4, 2019, to January 17, 2020. Time point 4 (T4) for cohort 1 was from September 10, 2019, to January 15, 2020, and for cohort 2 was February 23, 2021, to June 21, 2021.

Both cohorts showed deteriorations in mental health difficulties and mental well-being between T3 and T4 (Table 2 and Figure 2). However, cohort 2 (vs cohort 1) showed significantly greater deteriorations in risk for depression (adjusted mean difference [AMD], 1.91; 95% CI, 1.07-2.76; *g* = 0.17); social, emotional, and behavioral difficulties (AMD, 0.76; 95% CI, 0.33-1.18; *g* = 0.12); and mental well-being (AMD, −2.08; 95% CI, −2.80 to −1.36; *g* = −0.22), with small effects.

**Table 2.** Descriptive Data and Within/Between Cohort Outcome Analyses^a^

Status	Mean (SD)		AMD (95% CI)	*P* value	Hedges *g*	ICC	Mean (SD)		AMD (95% CI)	*P* value	Hedges *g*	ICC	Mean (SD)		AMD (95% CI)	*P* value	Hedges *g*	ICC
	CES-D T3	CES-D T4					SDQ T3	SDQ T4					WEMWBS T3	WEMWBS T4				
Cohort 1, prepandemic^b^	15.80 (11.66)	17.29 (12.04)	1.52 (0.84 to 2.20)	<.001	0.16	0.02	12.33 (6.70)	13.19 (6.62)	0.86 (0.53 to 1.20)	<.001	0.18	0.04	48.26 (9.49)	47.40 (9.39)	−0.85 (−1.45 to −0.24)	.006	−0.10	0.02
Cohort 2, pandemic^c^	16.80 (11.67)	20.26 (12.44)	3.43 (3.04 to 3.83)	<.001	0.33	0.02	12.69 (6.63)	14.32 (6.55)	1.62 (1.42 to 1.82)	<.001	0.30	0.02	47.83 (9.83)	44.87 (9.80)	−2.92 (−3.25 to −2.59)	<.001	−0.32	0.02
Between cohort	NA	NA	1.91 (1.07 to 2.76)	<.001	0.17	0.02	NA	NA	0.76 (0.33 to 1.18)	<.001	0.12	0.02	NA	NA	−2.08 (−2.80 to −1.36)	<.001	−0.22	0.02

Abbreviations: AMD, adjusted mean difference; CES-D, Center for Epidemiological Studies for Depression scale; ICC, intracluster correlation coefficient ; NA, not applicable; SDQ, Strengths and Difficulties Questionnaire; T3, time point 3; T4, time point 4; WEMWBS, Warwick-Edinburgh Mental Well-Being Scale.

^a^ Mixed-effects linear regressions with maximum likelihood estimation were fitted, including schools (clusters) as random effects and adjusted for the country, school size, coeducation, allocation group, and the time difference (days) between T3 and T4. Descriptive data are raw. The degree of school-level clustering, using the ICC for the 3 mental health outcomes, was evaluated. Unconditional means models were fitted, using 2-level mixed-effects linear regressions at T3 to estimate ICCs at the school level. All differences remained significant when the multiple testing correction was applied. The CES-D range is 6 to 60; SDQ, 0 to 40; and WEMWBS, 14 to 70. T3 for cohort 1 was from September 21, 2018, to February 14, 2019, and for cohort 2 was September 4, 2019, to January 17, 2020. T4 for cohort 1 was from September 10, 2019, to January 15, 2020, and for cohort 2 was February 23, 2021, to June 21, 2021.

^b^ In cohort 1, of 769 students providing data, 764 provided data on the CES-D, 763 on the SDQ, and 768 on the WEMWBS.

^c^ In cohort 2, of 2958 students providing data, 2934 provided data on the CES-D, 2903 on the SDQ, and 2948 on the WEMWBS.

### Associations With Changes in Students’ Mental Health and Well-Being

eTables 4 and 5 in Supplement 1 show the univariable models used to explore associations between student- and school-level characteristics and changes in students’ mental health and mental well-being over time in cohort 1 and cohort 2, respectively. eTable 6 and eFigures 3 and 4 in Supplement 1 show the results of the multivariable models for cohort 1.

In the pandemic-exposed cohort 2, students with a low and high initial risk for mental health difficulties showed deteriorations in the main outcomes. Deteriorations were greater in students with low (vs high) initial risk for mental health difficulties for risk for depression (B, 2.07; 95% CI, 0.82-3.32) and social, emotional, and behavioral difficulties (B, 0.99; 95% CI, 0.41-1.57) (Table 3^33^ and eTable 7 and eFigure 5 in Supplement 1). Over time, female (vs male) students were at an increased risk for depression (B, 1.64; 95% CI, 0.54-2.73) and social, emotional, and behavioral difficulties (B, 1.63; 95% CI, 1.09-2.17), as well as poorer mental well-being (B, −1.42; 95% CI, −2.41 to −0.43) (eFigure 6 in Supplement 1). In contrast, over time, stronger home connectedness was associated with a decreasing risk for depression (B, −8.95; 95% CI, −11.40 to −6.51) and social, emotional, and behavioral difficulties (B, −3.66; 95% CI, −4.72 to −2.60), as well as increased well-being (B, 6.24; 95% CI, 4.21-8.27) (eFigure 7 in Supplement 1). A more positive student-rated school climate at T3 was also associated with a decreasing risk for depression (B, −4.47; 95% CI, −5.17 to −3.78) and social, emotional, and behavioral difficulties (B, −2.76; 95% CI, −3.12 to −2.40), as well as increased well-being (B, 3.90; 95% CI, 3.35-4.46), but the association between school climate and outcomes weakened over time (risk for depression: B, 2.68; 95% CI, 1.91-3.44; social, emotional, and behavioral difficulties: B, 1.54; 95% CI, 1.19-1.89; and well-being: B, −2.55; 95% CI, −3.19 to −1.91) (eFigure 8 in Supplement 1). Uncertainty around having a friend during lockdown was associated with an increased risk for depression (B, 2.88; 95% CI, 0.63-5.14) and social, emotional, and behavioral difficulties (B, 1.25; 95% CI, 0.21-2.28) (eFigure 9 in Supplement 1). Household assets, studying conditions, home conflicts, and how much time students were in school during lockdown were not significantly associated with changes in mental health.

**Table 3.** Multivariable Analyses for Risk for Depression; Social, Emotional, and Behavioral Difficulties; and Mental Well-Being in Cohort 2^a^

Student characteristics	CES-D		SDQ		WEMWBS	
	B (95% CI)	*P* value	B (95% CI)	*P* value	B (95% CI)	*P* value
Age	1.25 (0.21 to 2.29)	.02	NA	NA	−0.59 (−1.36 to 0.18)	.13
Interaction between time and age	−0.29 (−1.16 to 0.58)	.51	NA	NA	0.61 (−0.12 to 1.34)	.10
Gender						
Female (vs male)	4.78 (3.71 to 5.84)	<.001	1.26 (0.70 to 1.82)	<.001	−2.99 (−3.86 to −2.12)	<.001
Other/prefer not to say (vs male)	5.56 (2.47 to 8.66)	<.001	1.25 (−0.45 to 2.96)	.15	−4.40 (−6.85 to −1.95)	<.001
Interaction between time and female	1.64 (0.54 to 2.73)	.003	1.63 (1.09 to 2.17)	<.001	−1.42 (−2.41 to −0.43)	.005
White race (vs other racial and ethnic groups^b^)	NA	NA	1.20 (0.70 to 1.70)	<.001	NA	NA
Year group 10 (vs year group 9)	−1.35 (−2.61 to −0.08)	.04^c^	0.19 (−0.31 to 0.69)	.46	0.09 (−0.74 to 0.93)	.83
Interaction between time and year group 10	NA	NA	−0.26 (−0.73 to 0.22)	.29	NA	NA
Student-rated school climate	−4.47 (−5.17 to −3.78)	<.001	−2.76 (−3.12 to −2.40)	<.001	3.90 (3.35 to 4.46)	<.001
Interaction between time and student-rated school climate	2.68 (1.91 to 3.44)	<.001	1.54 (1.19 to 1.89)	<.001	−2.55 (−3.19 to −1.91)	<.001
Low risk for mental health difficulties (vs high)	−5.96 (−7.10 to −4.82)	<.001	−3.72 (−4.31 to −3.13)	<.001	3.30 (2.40 to 4.21)	<.001
Interaction between time and low risk for mental health difficulties	2.07 (0.82 to 3.32)	.001	0.99 (0.41 to 1.57)	.001	−0.93 (−1.98 to 0.12)	.08
Home environment						
Household assets	−1.51 (−3.85 to 0.84)	.21	−0.53 (−1.63 to 0.57)	.34	0.32 (−1.27 to 1.90)	.69
Interaction between time and household assets	2.50 (−0.02 to 5.02)	.05	NA	NA	NA	NA
Studying conditions	−1.58 (−4.26 to 1.10)	.25	−1.31 (−2.53 to −0.09)	.04^c^	0.94 (−1.21 to 3.08)	.39
Interaction between time and studying conditions	−1.03 (−3.96 to 1.90)	.49	NA	NA	0.34 (−2.10 to 2.78)	.78
Home connectedness	−12.09 (−14.35 to −9.83)	<.001	−5.83 (−6.99 to −4.67)	<.001	9.66 (7.87 to 11.45)	<.001
Interaction between time and home connectedness	−8.95 (−11.40 to −6.51)	<.001	−3.66 (−4.72 to −2.60)	<.001	6.24 (4.21 to 8.27)	<.001
Home conflicts						
Sometimes (vs lots of times)	0.05 (−1.21 to 1.31)	.94	0.36 (−0.32 to 1.05)	.30	−0.62 (−1.59 to 0.35)	.21
Rarely	0.33 (−1.08 to 1.74)	.65	−0.27 (−1.01 to 0.48)	.48	−0.46 (−1.57 to 0.64)	.41
No/don’t know	−0.80 (−2.16 to 0.56)	.25	−0.80 (−1.52 to −0.09)	.03^c^	0.34 (−0.73 to 1.41)	.53
Interaction between time and home conflicts rarely	−0.16 (−1.51 to 1.19)	.82	0.22 (−0.40 to 0.84)	.49	0.05 (−1.07 to 1.17)	.93
Interaction between time and home conflicts no/don’t know	−1.01 (−2.28 to 0.26)	.12	−0.15 (−0.73 to 0.43)	.60	0.32 (−0.74 to 1.39)	.56
Friendships						
Don’t know (vs yes)	2.20 (0.13 to 4.26)	.04^c^	0.32 (−0.75 to 1.38)	.56	−2.57 (−3.90 to −1.25)	<.001
No (vs yes)	3.03 (1.49 to 4.57)	<.001	1.98 (1.14 to 2.82)	<.001	−2.76 (−3.99 to −1.54)	<.001
Prefer not to say (vs yes)	1.95 (−1.33 to 5.23)	.24	2.06 (0.33 to 3.78)	.02	−2.62 (−5.10 to −0.14)	.04^c^
Interaction between time and friendships, don’t know	2.88 (0.63 to 5.14)	.01	1.25 (0.21 to 2.28)	.02	NA	NA
**Operational features of the school**						
School size	0.0001 (−0.003 to 0.003)	.97	NA	NA	NA	NA
Interaction between time and school size	−0.0002 (−0.002 to 0.002)	.87	NA	NA	NA	NA
Coeducational (vs female only)	1.11 (0.06 to 2.15)	.04^c^	0.12 (−0.55 to 0.79)	.72	−0.44 (−1.50 to 0.63)	.42
Interaction between time and coeducational	NA	NA	0.16 (−0.43 to 0.74)	.60	−0.10 (−1.27 to 1.07)	.87
School quality						
Requires improvement (vs outstanding)	0.02 (−2.71 to 2.74)	.99	NA	NA	NA	NA
Interaction between time and school quality, requires improvement	−2.51 (−5.43 to 0.40)	.09	NA	NA	NA	NA
SEL quality rating	NA	NA	−0.09 (−0.23 to 0.05)	.22	0.03 (−0.21 to 0.26)	.82
Interaction between time and SEL quality rating	NA	NA	NA	NA	0.07 (−0.14 to 0.28)	.52
Some time at school during lockdown (vs at home)	−0.57 (−2.27 to 1.12)	.51	0.58 (−0.31 to 1.48)	.20	0.87 (−0.43 to 2.17)	.19
Student-rated school climate	−5.47 (−8.24 to −2.71)	<.001	−1.26 (−2.78 to 0.26)	.11	2.60 (0.31 to 4.90)	.03^c^
**Characteristics of the school community**						
% SEND support	NA	NA	0.03 (−0.03 to 0.08)	.35	NA	NA
Students’ age (school level)	−0.32 (−2.55 to 1.90)	.78	NA	NA	NA	NA
Interaction between time and students’ age	2.18 (0.24 to 4.12)	.03^c^	NA	NA	NA	NA
% Students who are White^d^	NA	NA	NA	NA	−0.01 (−0.03 to 0.004)	.16
Interaction between time and % students who are White	NA	NA	NA	NA	0.001 (−0.02 to 0.02)	.94
Broader context						
Urbanicity rural (vs urban)	NA	NA	NA	NA	0.78 (−0.40 to 1.95)	.20
Area-level deprivation by IMD	NA	NA	NA	NA	0.02 (−0.10 to 0.14)	.73

Abbreviations: CES-D, Center for Epidemiological Studies for Depression scale; IMD, Indices of Multiple Deprivation; NA, not applicable; SDQ, Strengths and Difficulties Questionnaire; SEL, social-emotional learning; SEND, special educational needs or disabilities; WEMWBS, Warwick-Edinburgh Mental Well-Being Scale.

^a^ Multivariable analyses using multilevel linear regressions via maximum likelihood estimation and 3-level mixed-effects models for the analysis of the unique associations between student- and school-level characteristics and changes in students’ mental health and mental well-being between time point 3 (T3; September 4, 2019, to January 17, 2020) and time point 4 (T4; February 23, 2021, to June 21, 2021) during the COVID-19 pandemic, entering those factors that provided significant *P* values (*P* < .05) in the univariable analyses (eTable 5 in Supplement 1). The continuous student-level factors (age, school climate) were group mean (school level) centered; therefore, the regression coefficients represent an estimate of the differences in individual effects within schools. The continuous school-level factors were introduced as group means so that these regression coefficients represent school-level effects (ie, differences between schools).^33^ Regression coefficients of the interaction terms reflect changes relative to the first assessment (ie, T4 vs T3). All models controlled for design variables, trial allocation group, and the time difference (days) between T3 and T4. Variables that did not show significant univariable associations in any of the outcome variables are omitted.

^b^ The other racial and ethnic groups category includes Arab, Asian, Black/African/Caribbean, mixed/multiple ethnic groups, and other ethnic groups. This category was grouped together owing to small sample sizes.

^c^ This association was no longer significant when the multiple testing correction was applied.

^d^ Ford et al^12^ suggest a potential association of this variable at the school level with mental health outcomes at the student level.

Whether home connectedness at T4 was associated with student-rated school climate at T3 and T3 to T4 changes in mental health difficulties and mental well-being were explored in cohort 2 (eTable 8 in Supplement 1). The combination of poor student-rated school climate (T3) with high home connectedness (T4) was associated with the greatest (T3-T4) reduction in risk for depression. Conversely, the combination of good student-rated school climate and low home connectedness was associated with the greatest increase in risk for depression (eTable 9 and eFigure 10 in Supplement 1).

### Associations With Students’ Adjustment to Lockdown and Return to School

eTables 10 and 11 in Supplement 1 summarize the univariable and multivariable analyses of factors associated with students’ adjustment to lockdown and going back to school, respectively, in cohort 2. In the multivariable analysis, only higher home connectedness was associated with better adjustment during lockdown (B, 0.76; 95% CI, 0.50-1.02). On return to school, female gender was associated with worse adjustment (B, −0.28; 95% CI, −0.39 to −0.16), while higher prepandemic student-rated school climate (B, 0.24; 95% CI, 0.15-0.33), greater home connectedness (B, 0.53; 95% CI, 0.28-0.79), and partial school attendance during lockdown (B, 0.42; 95% CI, 0.20-0.63) were associated with better adjustment (for a summary of all associations, see eFigure 11 in Supplement 1).

## Discussion

During normal times, secondary school students have to navigate a period of growing and complex demands.^34,35,36,37^ Over the past decade, anxiety, depression, and self-harm have increased among this population, especially among girls.^1^ These difficulties in young peoples’ mental health increased during the COVID-19 pandemic.^5,8,10,34,35^ Consistent with earlier studies, the present study demonstrates small but relevant deteriorations in secondary students’ mental health difficulties and mental well-being over time. Herein, we show, to our knowledge, for the first time in a natural experiment from a single study that these deteriorations were exacerbated by the COVID-19 pandemic. The consistency of these results across different measures suggests that effects are real and meaningful. Previous research has shown that social isolation during the pandemic negatively influenced students’ mental health.^7^ We identified key individual (eg, gender), home (eg, connectedness), friendship (eg, friendships during lockdown), and school (eg, climate) factors that were associated with changes in students’ mental health.

Among those exposed to the pandemic, not only girls,^10^ but also students without perceived friendships during lockdown and those with a low initial risk for mental health difficulties were more likely to experience deteriorating mental health. Higher levels of student-rated school climate were associated with protection against such deteriorating mental health. A 2018 systematic review suggested that positive school climate is associated with better student mental health, but most studies were cross sectional.^38^ Although losing a supportive school environment amid the pandemic restrictions may have heightened home or community challenges, the association between school climate and mental health also weakened over time in students not exposed to the pandemic. This implies that the protective effect of a good school climate observed cross sectionally might decrease over time for all, perhaps due to academic pressures and uncertainties regarding their future, as the time to leave school draws closer. The effect of school-based interventions that promote a positive school climate on students’ mental health and mental well-being warrants further research.^38^

The present findings suggest that positive home connectedness protected against deteriorating mental health during the pandemic. Previous research observed that conflict with parents was associated with worse mental health during the pandemic,^8^ which we did not replicate here. A more complex picture emerged in terms of risk for depression. For students who had a poor school climate prepandemic and then spent time in a highly connected home environment, the pandemic was associated with improved outcomes for them. However, for students who had a more positive prepandemic school climate but were forced to stay at home in a poorly connected environment, the pandemic was associated with worsened outcomes. The combination of both positive environments (school climate and home connectedness) might contribute to maintaining a stable low risk for depression. These findings suggest that positive home and school environments could work additively and should be targeted together by policies and interventions.^39^

Only high home connectedness was significantly associated with greater adjustment to lockdown, consistent with previous research.^8^ Interestingly, higher student-rated school climate, positive home connectedness, and having been in school some of the time during lockdown were all associated with better adjustment when returning to school. Girls were more likely than boys to report challenges with returning to school. These findings suggest that schools should be kept open whenever possible. If reduced interaction is required, it is important to encourage children to attend school for some of the time. Attendance for students facing challenges, such as social isolation, needs to be prioritized. Future research should explore why girls were more likely to struggle when schools reopened. In summary, these findings extend earlier work by demonstrating the importance of supportive relationships and underscore the potential for family, peer, and school interventions to support the mental health of students.^39,40^

### Strengths and Limitations

Previous research used cohorts from different studies, including distinct interventions and a limited number of factors.^10^ We analyzed data from a large cluster randomized clinical trial focusing on a representative sample of UK secondary schools and students. Participants were recruited in 2 cohorts from the same study, providing a unique opportunity to test risk and resilience factors underpinning changes in students’ mental health during a critical developmental stage and a challenging period. While data were collected using mostly validated measures, some of them had not been used before (eg, the adjustment to lockdown and return to school variables), given the lack of existing measures. We conducted rigorous data analyses to explore a range of theory-driven characteristics across multiple levels of analysis.^12,13,14^ School-level characteristics were measured prepandemic as preexisting conditions to explore longitudinal associations with students’ mental health difficulties and mental well-being during the pandemic. However, some of these school-level features could have changed because of the pandemic (eg, the percentage of students eligible for free school meals in England for year 10 increased 12% between 2019-2020 and 2020-2021),^41^ which await further scrutiny. The present sample was majority White, and subgroup analyses for other racial and ethnic minority groups were impossible due to small numbers. While we did not have information on the socioeconomic status of each student, we used characteristics of the home environment during lockdown, such as household assets and studying conditions, as a proxy. Other possible influences on students’ adaptation (eg, trauma) await future research. No clinical indicators of mental health diagnoses were available; hence, we relied on student self-report. The home environment and friendship measures were added at the final follow-up to collect information retrospectively, risking recall bias.^42^ Although students with and without follow-up data were similar, the attrition rate was high in cohort 2 owing to the challenges of engaging schools and students during partial lockdown.^10^

## Conclusions

In this cohort study, across the broader context of deteriorating mental health in UK students, we observed that the COVID-19 pandemic considerably exacerbated this trend. We also identified individual, home, friendship, and school characteristics that were associated with changes in mental health difficulties and mental well-being. These findings have policy implications, suggesting that students’ risk and resilience should be conceptualized as multidimensional, including individual, family, friendship, and school factors. Future interventions need to consider all of these levels to effectively address the increasing mental health challenges faced by secondary school students.

## Notes

Supplement 1. **eFigure 1.** Pandemic timeline, lockdown restrictions at T4 (Cohort 2), and participating students according to restrictions
eMethods.
**eTable 1.** Missing data and post hoc power calculation **eTable 2.** Representativeness of included (T3-T4) schools and students by cohort **eTable 3.** Descriptive data of students’ home environment and adjustment during lockdown and return to school, added in Cohort 2 at T4 **eFigure 2.** Students’ transitions in terms of risk for depression, social-emotional-behavioral difficulties and mental well-being from T3 to T4 by cohort **eTable 4.** Univariable analyses for risk for depression, social-emotional-behavioral difficulties, and well-being (Cohort 1) **eTable 5.** Univariable analyses of risk for depression, social-emotional-behavioral difficulties, and well-being (Cohort 2) **eTable 6.** Multivariable analyses of risk for depression, social-emotional-behavioral difficulties, and well-being (Cohort 1) **eFigure 3.** Relationship between student-rated school climate and outcomes as a function of time (Cohort 1) **eFigure 4.** Relationship between coeducation and outcomes as a function of time (Cohort 1) **eTable 7.** Descriptive data and within-cohort outcome analyses by initial risk for mental health difficulties **eFigure 5.** Outcomes by initial risk for mental health difficulties, cohort status, and time point **eFigure 6.** Relationship between gender (female vs. male) and outcomes as a function of time (Cohort 2) **eFigure 7.** Relationship between home connectedness and outcomes as a function of time (Cohort 2) **eFigure 8.** Relationship between student-rated school climate and outcomes as a function of time (Cohort 2) **eFigure 9.** Relationship between friendship and outcomes as a function of time (Cohort 2) **eTable 8.** ‘Student-rated school climate (student level) x home connectedness x time’ three-way interaction in the analyses of risk for depression, social-emotional-behavioral difficulties, and well-being (Cohort 2) **eTable 9.** Multivariable analyses for risk for depression and social-emotional-behavioural difficulties, incorporating the “Time*Student-rated school climate*Home connectedness” three-way interaction (Cohort 2) **eFigure 10.** Relationship between student-rated school climate and risk for depression as a function of home connectedness **eTable 10.** Univariable and multivariable analyses of factors associated with adolescents’ adjustment to lockdown (Cohort 2) **eTable 11.** Univariable and multivariable analyses of factors associated with adolescents’ adjustment to return to school (Cohort 2) **eFigure 11.** Evidence map of the associations found in the present study
eReferences

Supplement 2. **Nonauthor Collaborators.** The MYRIAD Team

Supplement 3.
Data Sharing Statement
