# Supplementary material for: Young People’s Mental Health Changes, Risk, and Resilience During the COVID-19 Pandemic
Source: JAMA Netw Open. 2023 Sep 21;6(9):e2335016. doi: 10.1001/jamanetworkopen.2023.35016 (PMC10514742; doi:10.1001/jamanetworkopen.2023.35016)
Supplement: Supplement 1. — eFigure 1. Pandemic timeline, lockdown restrictions at T4 (Cohort 2), and participating students according to restrictions eMethods. eTable 1. Missing data and post hoc power calculation eTable 2. Representativeness of included (T3-T4) schools and students by cohort eTable 3. Descriptive data of students’ home environment and adjustment during lockdown and return to school, added in Cohort 2 at T4 eFigure 2. Students’ transitions in terms of risk for depression, social-emotional-behavioral difficulties and mental well-being from T3 to T4 by cohort eTable 4. Univariable analyses for risk for depression, social-emotional-behavioral difficulties, and well-being (Cohort 1) eTable 5. Univariable analyses of risk for depression, social-emotional-behavioral difficulties, and well-being (Cohort 2) eTable 6. Multivariable analyses of risk for depression, social-emotional-behavioral difficulties, and well-being (Cohort 1) eFigure 3. Relationship between student-rated school climate and outcomes as a function of time (Cohort 1) eFigure 4. Relationship between coeducation and outcomes as a function of time (Cohort 1) eTable 7. Descriptive data and within-cohort outcome analyses by initial risk for mental health difficulties eFigure 5. Outcomes by initial risk for mental health difficulties, cohort status, and time point eFigure 6. Relationship between gender (female vs. male) and outcomes as a function of time (Cohort 2) eFigure 7. Relationship between home connectedness and outcomes as a function of time (Cohort 2) eFigure 8. Relationship between student-rated school climate and outcomes as a function of time (Cohort 2) eFigure 9. Relationship between friendship and outcomes as a function of time (Cohort 2) eTable 8. ‘Student-rated school climate (student level) x home connectedness x time’ three-way interaction in the analyses of risk for depression, social-emotional-behavioral difficulties, and well-being (Cohort 2) eTable 9. Multivariable analyses for risk for depression an [file jamanetwopen-e2335016-s001.pdf]

Supplemental Online Content

Montero-Marin J, Hinze V, Mansfield K, et al. Young people's mental health changes, risk, and resilience during the COVID-19 pandemic. *JAMA Netw Open*. 2023;6(9):e2335016. doi:10.1001/jamanetworkopen.2023.35016

eFigure 1. Pandemic timeline, lockdown restrictions at T4 (Cohort 2), and participating students according to restrictions
eMethods.
eTable 1. Missing data and post hoc power calculation
eTable 2. Representativeness of included (T3-T4) schools and students by cohort
eTable 3. Descriptive data of students' home environment and adjustment during lockdown and return to school, added in Cohort 2 at T4
eFigure 2. Students' transitions in terms of risk for depression, social-emotional-behavioral difficulties and mental well-being from T3 to T4 by cohort
eTable 4. Univariable analyses for risk for depression, social-emotional-behavioral difficulties, and well-being (Cohort 1)
eTable 5. Univariable analyses of risk for depression, social-emotional-behavioral difficulties, and well-being (Cohort 2)
eTable 6. Multivariable analyses of risk for depression, social-emotional-behavioral difficulties, and well-being (Cohort 1)
eFigure 3. Relationship between student-rated school climate and outcomes as a function of time (Cohort 1)
eFigure 4. Relationship between coeducation and outcomes as a function of time (Cohort 1)
eTable 7. Descriptive data and within-cohort outcome analyses by initial risk for mental health difficulties
eFigure 5. Outcomes by initial risk for mental health difficulties, cohort status, and time point
eFigure 6. Relationship between gender (female vs. male) and outcomes as a function of time (Cohort 2)
eFigure 7. Relationship between home connectedness and outcomes as a function of time (Cohort 2)
eFigure 8. Relationship between student-rated school climate and outcomes as a function of time (Cohort 2)
eFigure 9. Relationship between friendship and outcomes as a function of time (Cohort 2)
eTable 8. 'Student-rated school climate (student level) x home connectedness x time' three-way interaction in the analyses of risk for depression, social-emotional-behavioral difficulties, and well-being (Cohort 2)
eTable 9. Multivariable analyses for risk for depression and social-emotional-behavioural difficulties, incorporating the "Time*Student-rated school climate*Home connectedness" three-way interaction (Cohort 2)
eFigure 10. Relationship between student-rated school climate and risk for depression as a function of home connectedness

eTable 10. Univariable and multivariable analyses of factors associated with adolescents' adjustment to lockdown (Cohort 2)
eTable 11. Univariable and multivariable analyses of factors associated with adolescents' adjustment to return to school (Cohort 2)
eFigure 11. Evidence map of the associations found in the present study **eReferences**

This supplemental material has been provided by the authors to give readers additional information about their work.

eFigure 1. Pandemic timeline, lockdown restrictions at T4 (Cohort 2), and participating students according to restrictions

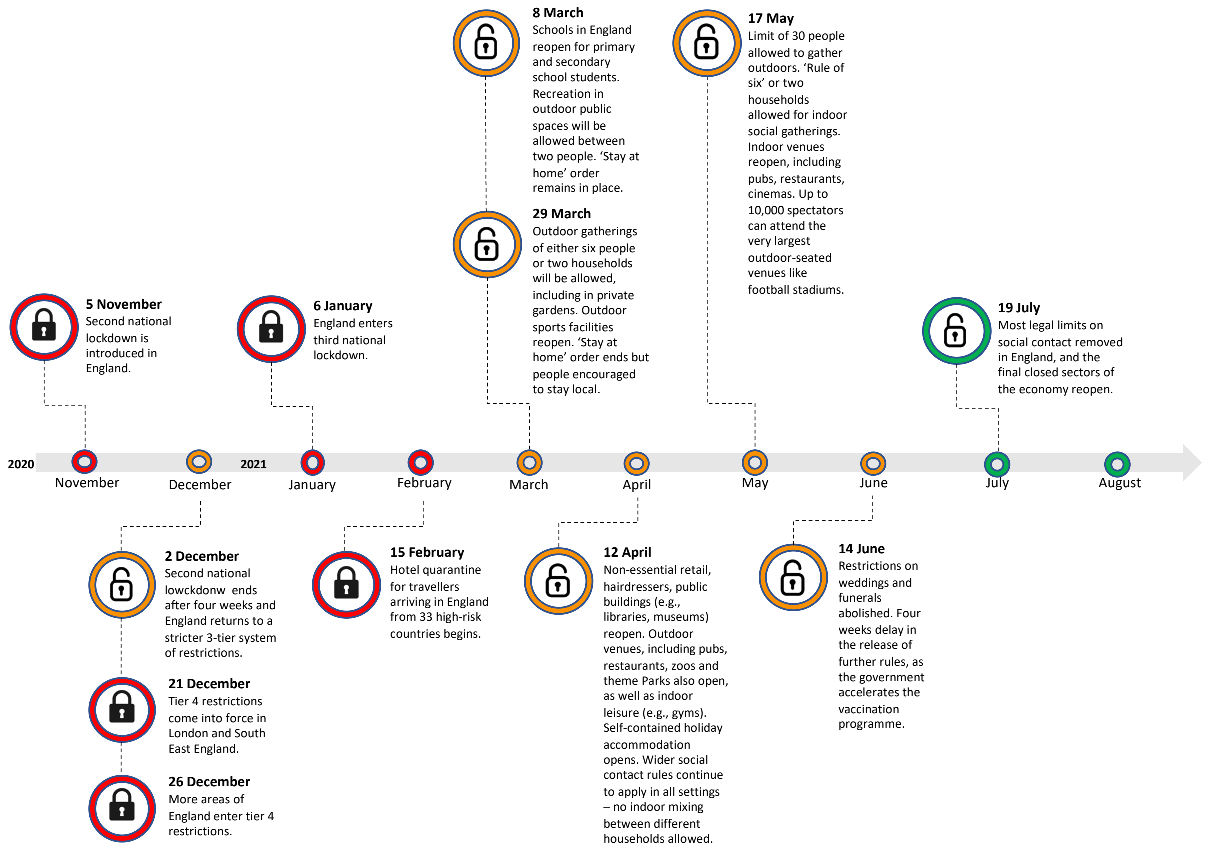

Timeline of students responding at T4 (Cohort 2) according to covid-19 restrictions

Time Windows (2021)	n	%	% accumulated
23 February – 7 March	1061	33.3	33.3
8 March – 11 April	576	18.1	51.4
12 April – 16 May	689	21.7	73.1
17 May – 13 Jun	703	22.0	95.1
14 Jun – 21 Jun	155	4.9	100

Total of students providing assent at T4 (Cohort 2): N = 3184

Source: Timeline of UK government coronavirus lockdowns and measures, March 2020 to December 2021.[1]

© 2023 Montero-Marin J et al. *JAMA Netw Open.*

eMethods.

Study measures

For Cohort 1, all data were collected in person. For Cohort 2, data for T3 were collected in person and data for T4 were collected online, in keeping with pandemic restrictions. All school-level factors were measured at T0, except teacher- and student-rated school climate, which were measured at T3. Cohort 2 students' adjustment to lockdown/return to school, home environment characteristics and friendships during lockdown were measured at T4 (a graphical description of the timeline of the measures is presented in Figure 1).

1. Students

1.1. Main outcomes

Risk for depression was measured using the "Centre for Epidemiologic Studies-Depression" (CES-D)[2] scale; a 20-item questionnaire (e.g., "I thought my life had been a failure") that assesses depressive symptoms, and is validated for use in adolescents. Each item is rated on a rating-scale from 0 ("rarely or none of the time" to 3 ("most or all of the time"), yielding a total score that ranges between 0 and 60, where higher scores indicate a higher risk for depression. The internal consistency (Cronbach's alpha (α)) of the CES-D total score in our study sample at T3 was α = 0.92).

Social-emotional-behavioral difficulties were measured using the "Strengths and Difficulties Questionnaire" (SDQ),[3] a 25-item questionnaire that assesses psychopathology over the previous 6 months and has been validated for use in adolescents. The 5 subscales assess emotional symptoms (e.g., "I worry a lot"), conduct problems (e.g., "I take things that are not mine from home, school or elsewhere"), hyperactivity/inattention (e.g., "I am easily distracted, I find it difficult to concentrate"), peer problems (e.g., "I fight a lot. I can make other people do what I want"), and prosocial behaviour (e.g., "I often volunteer to help others (parents, teachers, children"). Each item is rated on a rating-scale from 0 ("not true") to 2 ("certainly true"). We report a total score (range, 0–40) derived by summing the first 4 subscales, where higher scores indicate higher levels of social-emotional-behavioral difficulties. The internal consistency of the SDQ total score in our study sample at T3 was α = 0.85).

Mental well-being was measured using the "Warwick-Edinburgh Mental Well-Being Scale" (WEMWBS),[4] a 14-item measure assessing psychological well-being that is validated for use in adolescents.[5] Each item (e.g., "I've been able to make up my own mind about things") is scored on a rating-scale from 1 ("none of the time") to 5 ("all of the time"), yielding a total score between 14 and 70, where higher scores indicate greater levels of well-being. The internal consistency value of the WEMWBS total score in our study sample at T3 was α = 0.91).

1.2. Other outcomes

© 2023 Montero-Marin J et al. *JAMA Netw Open.*

The **adjustment to** the changing circumstances (i.e., **lockdown** and **return to school**), resulting from the Covid-19 pandemic, on the student's life was measured using the following two items that were designed for the purpose of the present study, and that were considered independently: a) "For some people, the lockdown made their lives better and for others it has made it worse. How did lockdown affect your life?"; and b) "For some people, going back to school after lockdown has made their lives better and for others it has made it worst. How has the return to school after lockdown affected your life?". Both (single item) questions were answered on a 5-point Likert-type scale that ranged from 1 ("life was much worse") to 5 ("life was much better").

1.3. Explanatory factors

Student factors (measured at T3) included **age** (in years), **gender identity** (male, female, other/prefer not to say) and **self-classified ethnicity** (using the following options defined by the research team: White, Arab, Asian, Black/African/Caribbean, mixed/multiple ethnic groups, other ethnic groups). Due to the small numbers observed in our sample and to facilitate data analyses, we recoded this variable as 'White' and 'other ethnic groups' (which included Arab, Asian, Black/African/Caribbean, mixed/multiple ethnic groups, other ethnic groups). We categorized students' ethnicity in this way as 'White' vs 'other ethnic groups' can be significantly related to mental health outcomes in adolescence.[6] We measured the student's **year group** across the home nations as follows: England 9 & 10; Northern Ireland 10 & 11; Scotland S2 & S3.

The **student-rated school-climate** (measured at T3) was assessed using the "Alaska School Climate and Connectedness Survey" (SCCS).[7] The SCCS student version measures aspects of school climate (e.g., social and environmental factors that contribute to the subjective experience of a school), and connectedness (e.g., perceptions and feelings about the people at school) for students, asking them to consider the way the school is "most of the time". The SCCS scale includes 40 items (7 subscales), all with 5-point Likert responses (from 1= "strongly agree" to 5 = "strongly disagree"). For the current study, 21 questions from the original SCCS questionnaire were employed, including the 'School Leadership and Student Involvement' (e.g., "At school, decisions are made based on what is best for students"), 'Respectful Climate' (e.g., "My teachers treat me with respect"), 'Peer Climate' (e.g., "Students in this school help each other, even if they are not friends"), and 'Caring Adults' (e.g., "There is at least one adult at this school whom I feel comfortable talking to about things that are bothering me") sub-scales. We used total scores which were calculated by summing the corresponding subscale scores divided by the number of items, with higher scores representing a better school climate (range: 1-5). The internal consistency of the student rated school-climate variable (SCCS total score) in our study sample at T3 was $\alpha = 0.92$.

The **risk for mental health difficulties** variable was defined by Latent Profile Analysis (LPA) reflecting subgroups of children with particular baseline (T0) patterns of risk for

mental health difficulties based on student characteristics (age, gender, ethnicity, social-emotional-behavioral difficulties, risk for depression, and well-being), the school's broader context (school urbanity), school community (school deprivation), and school operational features (school social-emotional learning (SEL) ethos).[6] LPA was developed in three steps and was conducted using maximum likelihood estimation with cluster (students within schools) robust standard errors. We were interested in classes that are optimally separated and are more likely to reflect 'true' classes in the population, rather than in the full spectrum of heterogeneity. Therefore, we evaluated a series of LPA models containing one to eight latent profiles in a randomly selected sub-sample (split-half). To validate the structure of the selected latent profile model, we tested LPA models in the second half of the sample, and all subsequent analyses were then developed with the total sample. For model selection, we used the Akaike information criterion (AIC), consistent Akaike information criterion (CAIC), bayesian information criterion (BIC), sample-size-adjusted BIC (sBIC), Lo–Mendell–Rubin adjusted likelihood ratio test (LMR-LRT), as well as bootstrapped likelihood ratio test (BLRT), and we also calculated the index of classification accuracy (entropy).[8] All models were well identified, and more latent profiles resulted in lower values of these fit indices (AIC, CAIC, BIC, sBIC), and hence suggested a better model fit. However, the LMR-LRT identified only three profiles, and the best entropy value was obtained with two profiles. In addition, the Elbow Plot showed the steepest slope with only two profiles. Given all this information, we compared the two- and three-profile models for conceptual interpretability and clarity. The three-profile model replicated the higher risk profile of the two-profile model, and lower risk profile was split into two non-risk profiles. For a better balance between fit and parsimony, and to aid interpretation, we chose the two-profile model. To validate the structure of the selected two-profile model, we repeated the process with the validation sample, which replicated our findings and supported the validity of our two-profile model. Following confirmation of a two-profile model structure from the two independent split-half samples, the dataset was recombined, and the same method of LPA was applied to the full sample. This allowed us to estimate the latent profile measurement model, generating weights that reflect individual profile membership, as well as the measurement error of the latent profile variable. Then, the latent profile variable was used as a factor in the subsequent auxiliary model (i.e., mixed effects linear regression). The largest subgroup of students (72.8%) was mainly characterized by lower values of risk for depression and social-emotional-behavioral difficulties, as well as higher values of mental well-being. Students in this subgroup were also younger, more often identified as males, and other ethnic backgrounds than Whites, had a higher SEL ethos, and were more often from rural areas. Students in this subgroup were much less likely to be at risk of suffering from mental health difficulties, and thus, this sub-group was labelled as "low risk". On the contrary, the other subgroup of students (27.2%) had higher values of risk for depression and social-emotional-behavioral difficulties, as well as lower values of mental well-being, and were older, more often identified as females and Whites, had a lower SEL ethos, and were more often from urban areas. Students in this subgroup were more likely to be at risk of suffering from mental health difficulties, and thus, this subgroup was labelled as "high risk". The mean values of the "low risk" subgroup were

© 2023 Montero-Marin J et al. *JAMA Netw Open.*

in the low category of risk for depression and social-emotional-behavioral difficulties, and in the medium category of mental well-being; while the mean values of the "high risk" subgroup were in the at-risk category of risk for depression and social-emotional-behavioral difficulties, and in the low category of mental well-being. We assigned students into their most likely profile based on BCH weights, using variables that reflected the measurement error of the latent profile variable.[9] This two-subgroup model, used to assign students into latent profiles, was characterized by high posterior probabilities for all latent profiles across both the total sample and the randomly selected subsamples, suggesting low classification error. For more details on this variable see Montero-Marin et al. (2022).[10]

2. Home Environment

2.1. Explanatory factors

Student characteristics of the home environment during lockdown (T4) included household assets, studying conditions, home connectedness and home conflicts.

Household assets were measured using five items from the "Family Affluence Scale" (FAS-III):[11] "Does your family own a car, van, or truck?" ("No" = 0, "Yes, one" = 1, "Yes, two or more" = 2), "Do you have your own bedroom for yourself?" ("No" = 0, "Yes" = 1), "How many computers does your family own?" ("None" = 0, "One" = 1, "Two" = 2, "More than two" = 3), "Does your family have a dishwasher?" ("No" = 0, "Yes" = 1), "How many bathrooms (rooms with a bath/shower or both) are there in your home?" ("None" = 0, "One" = 1, "Two" = 2, "Three or more" = 3). Items were re-scaled into a common range from 0 to 1, and then were summed and divided by the number of items to calculate a total score (range, 0–1), with higher scores reflecting more affluence. The internal consistency of the total scale (at T4) was $\alpha = 0.62$.

Studying conditions at home were assessed using six items (with a "yes" = 1 / "no" = 0 response) that asked about having a quiet space, desk, computer, internet access, regular help from the teacher, and help from a parent/carer. Specifically, students were asked: "During lockdown did you have adequate access to the following support / resources at home? …A quiet space for working or studying, …A desk, …A laptop, tablet, or computer you can work on, …Good internet access, …Regular help from your teacher, school, or college, …Help from a parent or carer". Responses were summed and divided by the number of questions to calculate a total score that ranged from 0 to 1, with higher scores reflecting better studying conditions at home. The internal consistency of the total score of the studying conditions scale (at T4) was $\alpha = 0.67$.

Home connectedness was measured using the "Family Connectedness Scale".[12] This questionnaire consist of six items that were re-scaled into a range from 0 to 1, and then summed and divided by the number of items to calculate a total score, with higher scores reflecting better home connectedness. The items that form this scale are the following:

© 2023 Montero-Marin J et al. *JAMA Netw Open.*

"Could you talk to a parent/caregiver about problems you were having?" ("Yes" = 1 / "No" = 0), "How much did you feel your household cared about you?" (from "Not at all" = 1, to "Very much" = 7), "How much did you feel your household cared about your feelings?" (from "Not at all" = 1, to "Very much" = 7), "How much did you feel your household understood you?" (from "Not at all" = 1, to "Very much" = 7), "How much did you feel your household had lots of fun together?" (from "Not at all" = 1, to "Very much" = 7), "How much did you feel your household respected your privacy?" (from "Not at all" = 1, to "Very much" = 7). The internal consistency of the home connectedness scale (at T4) was $\alpha = 0.92$.

Home conflicts were assessed using the following one-item question: "When parents or other adults in the house got into arguments with each other, others may have seen or heard what is going on. Did you see or hear one of these arguments?", which included the following response options: "yes, lots of times", "yes, sometimes", "yes, but rarely", "no/don't know".

3. Friendships

3.1. Explanatory factors

Friendships were measured at T4 using the following one-item question: "During lockdown did you have at least one friend who you could turn to for support?", which included the following response options: "yes", "don't know", "no", "prefer not to say". In order to capture the potential uncertainties around friendships during lockdown, when students were no longer able to see their peers at school, we included all response options as separate categories in the analyses.

4. School-level characteristics

School-level characteristics refer to the school community, operational features of the school, and broader school context.[6] Data were obtained by linking publicly available governmental data to the school's postcode, unless otherwise specified. We selected measures that were directly comparable across all four nations within the UK (England, Northern Ireland, Scotland, and Wales). Otherwise, we mapped existing measures onto their English equivalent (e.g., school quality ratings). We used pre-pandemic measures of school community characteristics, operational features of the school, and broader school context in our study because we were mainly interested in the potential longitudinal relationships between pre-pandemic school-level characteristics and students' mental health difficulties and mental well-being over time. In other words, we wanted to evaluate how differences in preexistent school-level characteristics could be associated with longitudinal change in our outcomes.

4.1. Explanatory factors: Characteristics of the school community

School community factors refer to characteristics of the student population at T0, including **school deprivation** (i.e., % of students eligible for free school meals: in England, children living in households on income-related benefits (such as universal credit) are eligible for free school meals, as long as their annual household income does not exceed £7,400 after tax, not including welfare payments. This is the same in Wales and Scotland, however in Northern Ireland it is set at £14,000 a year), the percentage of **students receiving support for special educational needs or disabilities** (SEND), and the percentage of **students self-classified as White** (all range from 0% to 100%).

4.2. Explanatory factors: Operational features of the school

Operational features of the school at T0 included the total **number of students** within a school, **student-to-teacher ratio**, and **coeducation** (coeducational school, or female-only school). The most recent official school inspection rating (Ofsted) at baseline was used to obtain an ordinal rating of **school quality**. As the approach to the measurement of school quality differed in public (independent schools) and private schools and across the nations, we mapped all school inspection rating systems onto the following categories: "requires improvements" = 0; "good" = 1; "outstanding" = 2.[13] **Quality of SEL provision** was assessed through a semi-structured interview with the senior leadership team or a staff member with overall responsibility for teaching SEL, using a list of 16 quality indicators, specifically designed for the original trial.[10] SEL in England is taught as part of 'Personal, Social, Health and Economic Education' (PSHE) lessons. Due to the fact that delivering PSHE lessons in schools is not mandatory in England, there is wide variation across schools in the delivery of PSHE lessons (in terms of content covered and teaching time allocated). For inclusion in the study, schools had to meet 5 criteria for their current PSHE provision: regular, discrete, named teaching time for PSHE (or equivalent); a designated PSHE lead; a named member of the Senior Leadership Team (SLT) responsible for PSHE; documentation denoting clear strategic planning of SEL within the school; and evaluation of pupil progress in PSHE. Once schools became a participating school, PSHE was assessed by discussing PSHE provision with the teacher responsible for PSHE at each school (or a member of the Senior Leadership Team). Sixteen quality indicators (see below) were used to assess PSHE provision. They were created specifically for this trial and identified through a review of existing measures and via expert consultation.[14] Schools were assigned a total score (a higher school rating (range: 0-16) indicates better SEL provision) reflecting the number of quality indicators present (in the following domains: "Leadership and Strategic Approaches to PSHE", "Curriculum Content and Delivery" and "Assessment, Evaluation, and Consultation"). The items used organised by their corresponding domain were the following:

Leadership and Strategic Approaches to PSHE from Consensus Indicators	A designated PSHE lead (0 = no, 1 = yes)<br>A named member of SLT has responsibility for supporting PSHE (0 = no, 1 = yes)<br>A written PSHE policy (0 = no, 1 = yes)<br>School's own rating of the quality of its PSHE provision (0- 4 = 0, 5-10 = 1)<br>PSHE provision is part of the school improvement plan (0 = no, 1 = yes)

© 2023 Montero-Marin J et al. *JAMA Netw Open.*

	How well-informed does the PSHE lead feel about local PSHE education CPD opportunities (0-4 = 0, 5-10 = 1)
Curriculum Content and Delivery from Consensus Indicators	Regular discrete, named teaching time for PSHE, including drop down days or tutorial time (0 = no, 1 = yes)<br>PSHE lead teaches PSHE lessons (0 = no, 1 = yes)<br>Topic Coverage KS3 and KS4 – School provides coverage of all elements of PSHE curriculum (0 = no, 1 = yes)<br>PSHE lead involved in planning: evidence of attempts to plan and coordinate PSHE across KS3 and KS4 (0 = no, 1 = yes)<br>Teaching Methods Used: School uses at least 6/10 methods for delivering PSHE (0 = no, 1 = yes)
Methods of Assessment, Evaluation and Consultation from Consensus Indicators	Any evaluation of pupil progress in PSHE (0 = no, 1 = yes) Informal feedback (0 = no, 1 = yes)<br>Pupil / peer assessment of feedback (0 = no, 1 = yes) Written feedback on pupil progress reports (0 = no, 1 = yes) School uses feedback to plan PSHE (0 = no, 1 = yes)

School SEL ethos (i.e., the underlying values and attitudes the school represents in relation to the way staff and students relate, the development of bonds between youth and adults, and the opportunities for participation in positive social activities),[15] was estimated by a new measure that evaluated the school's commitment to and progress towards mental health and well-being. This measure was developed by gathering existing data from various relevant sources at T0, identifying all those variables that map onto the hypothesized latent construct of school SEL ethos in relation to promoting students' social, emotional and mental well-being. The following school-level measures were considered: official school quality ratings (i.e., Ofsted),[13] average teacher-rated school climate (i.e., a school ecology total score measure aggregated from averaged teacher ratings based on the teacher version of the "School Climate and Connectedness Survey" that included the sub-scales of "School Leadership and Involvement", "Staff Attitudes" and "Respectful Climate"),[6] an assessment of PSHE provision (i.e, quality of SEL provision), and the school commitment to teaching SEL, rated by an independent evaluator and based on the direct observation of the school.[10] All these measures were re-scaled to a new range from 0 to 4 points to ensure that all the variables contributed equally to the computation of the final index. After this, Pearson's r correlations were calculated (range: from 0.22 to 0.58). Optimal implementation of parallel analysis was used as a dimensionality test to decide on the number of factors to be retained. The number of random correlation matrices used was 500 and the generation of random correlation matrices was based on the permutation of sample values. The advised number of dimensions was 1 when the mean of random percentage of variance was considered, which explained a total of 65% of real-data variance. The robust unweighted least squares (RULS) method, correcting for robust mean and variance adjusted chi-squared statistic, was employed for factor extraction, using the correlation matrix as data entry. The one-dimensional structure produced standardized loadings between 0.54 and 0.67. The factor determinacy index had a value of 0.85 and marginal reliability showed a value of 0.72. Construct replicability obtained a value of H = 0.72. The omega composite reliability for the unidimensional factor also obtained a value of 0.72. Factor scores were calculated by means of Bayes Expected a Posteriori –EAP– estimates transformed to T-scores, which ranged from 0 to 100, where higher scores represent a more conducive school ethos towards the promotion of social, emotional, and mental well-being. **School**

© 2023 Montero-Marin J et al. *JAMA Netw Open.*

attainment was obtained from publicly available governmental data, referring to the average attainment of students within a school. The attainment score is calculated based on the student's achievement across 8 subjects, including English and Mathematics (double weighted), and six further subjects (with a range between 9 and 90).[16-18] The SCCS was used to assess teacher-rated school climate.[6] A total score formed by the "School Leadership and Involvement" (e.g., "At school, decisions are made based on what is best for students"), "Staff Attitudes" (e.g., "Teachers and school staff believe that all students can do good work"), and "Respectful Climate" (e.g., "At this school, students and teachers get along really well") sub-scales was used, with higher scores representing a better school climate (range: 1-5). The internal consistency of the teacher-rated SCCS in our study sample at T3 was $\alpha = 0.92$. Total scores were calculated by taking the mean across teachers within a school to obtain a **school-level measure of teacher-rated school climate**. In addition, a measure of student-rated school climate at the school level (i.e., **school level student-rated school climate**) was also calculated following the same procedure (range: 1-5). The **time in school during the third lockdown** was measured using the following one-item question: "During the lockdown, did you…" ("stayed at home?", "attended school some of the time?", "still attended school full time?").

4.3. Explanatory factors: Broader school context

The broader school context summarises wider socioeconomic factors in the school's catchment area at T0 and includes the following variables: **urbanicity** (urban vs. rural school location) and **area-level deprivation** (Index of Multiple Deprivation 2015; IMD, decile rating (from "most deprived" = 1 to "least deprived" = 10)), which summarises deprivation across the categories of income, employment, health/disability, education/skills/training, crime, barriers to services/housing and living environment.[19]

© 2023 Montero-Marin J et al. *JAMA Netw Open.*

eTable 1. Missing data and post hoc power calculation

The initial study sample at T3 consisted of K=12 schools, N=864 students in Cohort 1 and K=72, N=6386 in Cohort 2. Of those, 12 schools and 769 students (89.0%) in Cohort 1 (pre-pandemic), and 54 schools and 2958 students (46.3%) in Cohort 2 (mid-pandemic), were retained until T4 and provided data on at least one outcome. Therefore, we observed a missing data rate of 11.0% of students in Cohort 1 and 53.7% of students in Cohort 2 (25.0% of schools in Cohort 2), with an overall non-response of 48.6% of students. The specific attrition numbers for each outcome can be seen in the footnote of the next table below (Selected characteristics of pupils included at T3 by T4 follow-up status and cohort). This table shows the differences between students retained and lost to follow-up at T4 in student characteristics. As can be seen, students who were retained (vs lost to follow-up) indicated marginally lesser mental health difficulties and greater well-being at T3, particularly in Cohort 1 compared to Cohort 2. As this study has more than 40% of missing data, we report the missing data patterns found and the results of the complete case analyses under the missing at random (MAR) assumption, recognizing that our analyses are exploratory.[20] Our exploration of the possible missing data mechanisms in the data set (see student characteristics included at T3 by T4 follow-up status and cohort below) showed that, in general, students with higher levels of mental health difficulties and lower levels of mental well-being at T3 were more likely to have missing data. Therefore, our results could be biased towards a more positive view of participating students' initial mental health and mental well-being. This could have affected Cohort 1 and Cohort 2 differently, as they likely showed different missing data patterns, reflecting the different circumstances under which measurements were carried out. In terms of the main aim (i.e., longitudinal cohort comparison), this is a limitation of this study. However, in terms of the secondary aims (i.e., longitudinal relationships between factors and outcomes in the mid-pandemic cohort), finding effects in our more conservative, healthier retained sample would suggest that results obtained are likely robust in the full Cohort 2 sample. To have a measure on how missingness might affect statistical power, we have developed a post hoc statistical power calculation (see below).

Selected characteristics of pupils included at T3 by T4 follow-up status and cohort

Pupil characteristics	Students lost to follow-up at T4*			Remaining students**		
	Cohort 1	Cohort 2	Total	Cohort 1	Cohort 2	Total
	**N = 95**	**N = 3428**	**N = 3523**	**N = 769**	**N = 2958**	**N = 3727**
**Age**, mean (SD)	13.6 (0.6)	13.7 (0.6)	13.7 (0.6)	13.6 (0.6)	13.6 (0.6)	13.6 (0.6)
**Gender**†						
Female, n (%)	53 (55.8)	1687 (50.4)	1740 (50.5)	424 (55.2)	1783 (61.3)	2207 (60.1)
Male, n (%)	40 (42.1)	1601 (47.8)	1641 (47.6)	327 (42.6)	1070 (36.8)	1397 (38.0)
Other / Prefer not to say, n (%)	2 (2.1)	62 (1.9)	64 (1.9)	17 (2.2)	54 (1.9)	71 (1.9)
**Ethnicity**†† – White, n (%)	77 (81.1)	2606 (78.2)	2683 (78.2)	631 (82.3)	2064 (71.1)	2695 (73.3)
**Year group**						
Year 9, n (%)	52 (54.7)	1817 (53.0)	1869 (53.1)	456 (59.3)	1791 (60.6)	2247 (60.3)
Year 10, n (%)	43 (45.3)	1611 (47.0)	1654 (46.9)	313 (40.7)	1167 (39.4)	1480 (39.7)
**Risk for depression**††† (CES-D), mean (SD)	16.8 (12.5)	17.2 (12.1)	17.2 (12.1)	15.8 (11.7)	16.8 (11.7)	16.6 (11.7)
**Social-emotional-behavioural difficulties** (SDQ) – self report††††, M (SD)	14.1 (6.5)	13.5 (7.0)	13.5 (7.0)	12.3 (6.7)	12.7 (6.6)	12.6 (6.6)
**Well-being** (WEMWBS)†††††, mean (SD)	46.2 (10.8)	47.3 (10.2)	47.2 (10.2)	48.3 (9.5)	47.8 (9.4)	47.9 (9.4)

* Defined as those pupils with missing data on all 3 primary outcomes at 2-year follow-up. ** Defined as those pupils with at least one of the 3 primary outcomes at 2-year follow-up.
† Sample size in lost to follow-up group: 3445: Cohort 1: 95; Cohort 2: 3350. Sample size in remaining students' group: 3675: Cohort 1: 768; Cohort 2: 2907.
†† Sample size in lost to follow-up group: 3429: Cohort 1: 95; Cohort 2: 3334. Sample size in remaining students' group: 3670: Cohort 1: 767; Cohort 2: 2903.
††† Sample size in lost to follow-up group: 3517: Cohort 1: 95; Cohort 2: 3422. Sample size in remaining students' group: 3721: Cohort 1: 767; Cohort 2: 2954.
†††† Sample size in lost to follow-up group: 3508: Cohort 1: 94; Cohort 2: 3414. Sample size in remaining students' group: 3717: Cohort 1: 766; Cohort 2: 2951.
††††† Sample size in lost to follow-up group: 3521: Cohort 1: 95; Cohort 2: 3426. Sample size in remaining students' group: 3723: Cohort 1: 769; Cohort 2: 2954.
CES-D: Center for Epidemiologic Studies for Depression Scale. SDQ: Strengths and Difficulties Questionnaire (Total Difficulties Score). WEMWBS: Warwick-Edinburgh Mental Well-Being Scale. School year groups correspond across the home nations as follows: England 9 & 10; Northern Ireland 10 & 11; Scotland S2 & S3.

© 2023 Montero-Marin J et al. *JAMA Netw Open.*

The sample size and power calculation were originally determined by the objectives of the main intervention trial (see protocol and update).[21,22] For the present study, we carried out a post hoc power calculation to assess whether changes in students' mental health difficulties (i.e., risk for depression and social-emotional-behavioral difficulties) and mental well-being from T3 to T4 differed by cohort status (i.e., Cohort 1 vs. Cohort 2). We used the following (observed) parameters for the calculation:

•Type I error: 0.05
•Test: Hotelling-Lawley Trace approach (this approach supports the inclusion of baseline covariates and uses the Wald test for the general linear mixed-effects model)[23]
•Factors:
  Cohort (Predictor)
  School (Cluster)
  CESD (Outcome)
  SDQ (Outcome)
  WEMWBS (Outcome)
  TIME (Repeated Measure)
  Covariate (Days between T3-T4)
•Means for outcome: CESD
  Cohort 1: (T3) = 15.80 and (T4) = 17.29
  Cohort 2: (T3) = 16.80 and (T4) = 20.26
•Means for outcome: SDQ
  Cohort 1: (T3) = 12.33 and (T4) = 13.19
  Cohort 2: (T3) = 12.69 and (T4) = 14.32
•Means for outcome: WEMWBS
  Cohort 1: (T3) = 48.26 and (T4) = 47.40
  Cohort 2: (T3) = 47.83 and (T4) = 44.87
•Outcome correlation:

	CESD	SDQ	WEMWBS
CESD	1	0.77	-0.76
SDQ	0.77	1	-0.64
WEBWBS	-0.76	-0.64	1

•Correlation for TIME: 0.6
•Intra class correlation for School cluster: 0.02
•Covariate correlation: 0.002, 0.056, 0.011, 0.045, 0.013, -0.063
•Relative group sizes for Cohort: 1:4.5
•Hypothesis Effect: Cohort x TIME
•Hypothesis nature: Between x Within
•Sampling Unit: a mean of 57 students in each school (which totals 3762 students)
•Sample Size details: Cohort 1 = 12 schools; Cohort 2 = 54 schools; Total = 66 schools

Under all these conditions, the statistical power obtained was 0.90, which means that our sample is powered to detect small effects (observed Hedges' g ranging from 0.12 to 0.22 in absolute value) for the main study aim. Maintaining these conditions but reducing the sample size to 54 schools (in Cohort 2, for the secondary aim), provides a statistical power of 0.87. This means that our sample was still adequately powered to detect small effects in the univariable regression analyses. As the multivariable regression analyses included factors that were significant in the univariable analyses, we assume that the statistical power for the multivariable analyses would even increase. By including those factors, we are able to capture more potential sources of variation, which reduces residual variance and can increase the model's ability to detect significant relationships.[24]

eTable 2. Representativeness of included (T3-T4) schools and students by cohort

Schools	Target [a,b]	Baseline	Cohort 1 (T3-T4)	Cohort 2 (T3-T4)
**Country**, n (%)				
England	68 (80)	75 (88)	12 (100)	46 (85)
Wales	5 (6)	3 (4)	0 (0)	2 (4)
Scotland	8 (9)	3 (4)	0 (0)	3 (6)
Northern Ireland	4 (5)	4 (5)	0 (0)	3 (6)
**Coeducation**, n (%)				
Female-only	9 (11)	12 (14)	1 (8)	10 (23)
Coeducational	76 (89)	73 (86)	11 (92)	44 (77)
**FSM**, n (%)				
Above average	21 (25)	29 (34)	3 (25)	23 (43)
Below average	64 (75)	56 (66)	9 (75)	31 (57)
**OFSTED**, n (%)				
Outstanding/Excellent/Very good	20 (24)	17 (20)	3 (25)	12 (22)
Good/Satisfactory	47 (55)	46 (54)	7 (58)	26 (48)
Requires improvement/Adequate	14 (16)	11 (13)	2 (17)	5 (9)
Not yet rated/NA/UNK	4 (5)	11 (13)	0 (0)	11 (20)
**Type of school**, n (%)				
Non-selective	74 (87)	74 (87)	11 (92)	45 (83)
Selective	4 (5)	8 (9)	1 (8)	6 (11)
Independent	7 (8)	3 (4)	0 (0)	3 (5)
**Size of school**, n (%)				
Small (<1000)	45 (53)	39 (46)	7 (58)	26 (48)
Large (>1000)	40 (47)	46 (54)	5 (42)	28 (52)

Students	Normative	Baseline	Cohort 1 (T3-T4)	Cohort 2 (T3-T4)
Gender, female, n (%)	(49.0) [c]	4509 (55)	424 (55)	1783 (61)
Ethnicity, white, n (%)	(77.3) [c]	6202 (76)	631(82)	2064 (71)
Risk for depression, M (SD)	13.9 (9.7) [d]	13.5 (9.9)	15.8 (11.6)	16.8 (11.7)
Social-emotional-behavioural difficulties, M (SD)	10.3 (5.2) [e]	11.8 (6.5)	12.3 (6.7)	12.7 (6.6)
Well-being, M (SD)	48.8 (6.8) [f]	49.7 (9.7)	48.3 (9.5)	47.8 (9.4)

FSM: free school meals. OFSTED: Office for Standards in Education, Children's Services and Skills. [a]Kuyken et al., (2017).[21] [b]Kuyken et al., (2022).[25] [c]Data obtained using online publicly available data published by the education and statistics departments (e.g., Department of Education, 2020; https://gov.uk).[26] All available data were collected according to its proximity to the year in which participating pupils provided baseline (T0) questionnaire data. [d]Briere et al. (2013).[27] [e]https://www.sdqinfo.org/norms/UKNorm3.pdf.[28] [f]Clarke et al. (2011).[5]

© 2023 Montero-Marin J et al. *JAMA Netw Open.*

eTable 3. Descriptive data of students' home environment and adjustment during lockdown and return to school, added in Cohort 2 at T4

**Variables**	
**Household assets**, *M (SD), range: 0 (low) – 1 (high)*	0.75 (0.22)
**Studying conditions**, *M (SD), range: 0 (low) – 1 (high)*	0.88 (0.19)
**Home connectedness**, *M (SD), range: 0 (low) – 1 (high)*	0.72 (0.26)
**Home conflicts**, *n (%)*	
Yes, lots of times	480 (18.1)
Yes, sometimes	544 (20.5)
Yes, but rarely	639 (24.0)
No / Don't know	995 (37.5)
**Friend during lockdown**, *n (%)*	
Yes	2235 (84.1)
Don't know	181 (6.8)
No	189 (7.1)
Prefer not to say	53 (2.0)
**During lockdown**, *n (%)*	
Stayed at home	2221 (83.5)
Attended school some of the time	200 (7.5)
Still attended school full time	240 (9.0)
**How did lockdown affect you**, M *(SD), range: 1 (life was worse) – 5 (life was better)*	2.95 (1.27)
**Going back to school**, *M (SD), range: 1 (life was worse) – 5 (life was better)*	2.96 (1.19)

A total of 2662 students provided data on 'household assets', 2636 students provided data on 'home connectedness', 2658 students provided data on 'home conflicts', 2127 students provided data on 'studying conditions', 2661 students provided data on 'how did lockdown affect you', 2662 students provided data on 'going back to school', 2661 students provided data on 'during lockdown', 2658 students provided data on 'friend during lockdown'.

eFigure 2. Students' transitions in terms of risk for depression, social-emotional-behavioral difficulties and mental well-being from T3 to T4 by cohort

Cut-off scores are based on the official scoring guidelines.[2-5] Depression: low (0–15); at risk of depression (16–27); caseness (28–60). Social-emotional-behavioral difficulties: normal (0–14); borderline (15–17); high (18–19); very high (20–40). Well-being: probable mental health difficulties (0-40); possible mental health difficulties (41-44); average mental health (45-59); high well-being (60-70).

2.1 Risk for depression

Cohort 1

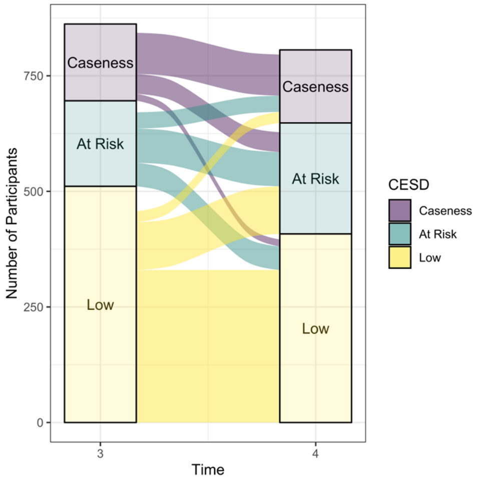

Cohort 2

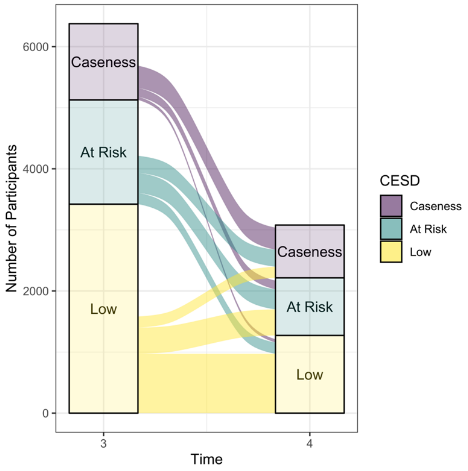

	Number (%)			Number (%)	
CESD	T3	T4	CESD	T3	T4
Caseness	166 (19.3%)	158 (19.6%)	Caseness	1249 (19.6%)	864 (28.1%)
At Risk	185 (21.5%)	240 (29.8%)	At Risk	1706 (26.8%)	943 (30.6%)
Low	511 (59.3%)	408 (50.6%)	Low	3421 (53.7%)	1272 (41.3%)

© 2023 Montero-Marin J et al. *JAMA Netw Open.*

2.2 Social-emotional-behavioral difficulties

Cohort 1

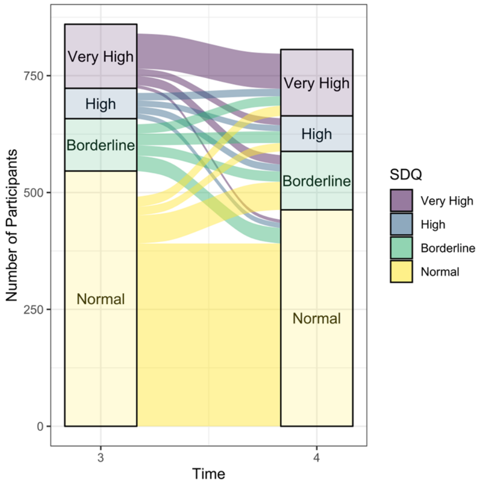

SDQ	Number (%) T3	T4
Very High	137 (15.9%)	142 (17.6%)
High	65 (7.6%)	76 (9.4%)
Borderline	112 (13.0%)	125 (15.5%)
Normal	546 (63.5%)	463 (57.4%)

Cohort 2

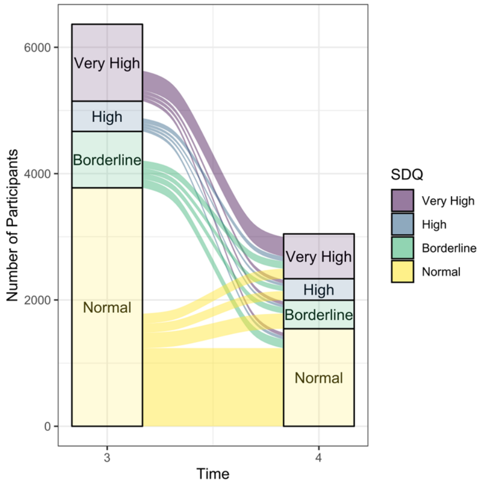

SDQ	Number (%) T3	T4
Very High	1218 (19.1%)	710 (23.3%)
High	479 (7.5%)	340 (11.2%)
Borderline	893 (14.0%)	452 (14.8%)
Normal	3775 (59.3%)	1544 (50.7%)

2.3 Mental well-being

Cohort 1

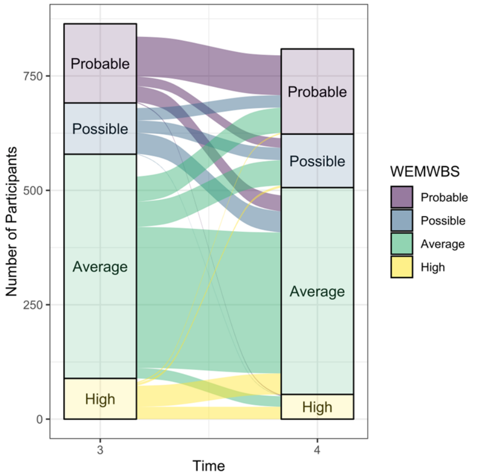

WEMWBS	Number (%) T3	T4
Probable MH Difficulties	173 (20.0%)	186 (23.0%)
Possible MH Difficulties	112 (13.0%)	117 (14.5%)
Average MH	490 (56.7%)	452 (55.9%)
High Wellbeing	89 (10.3%)	54 (6.7%)

Cohort 2

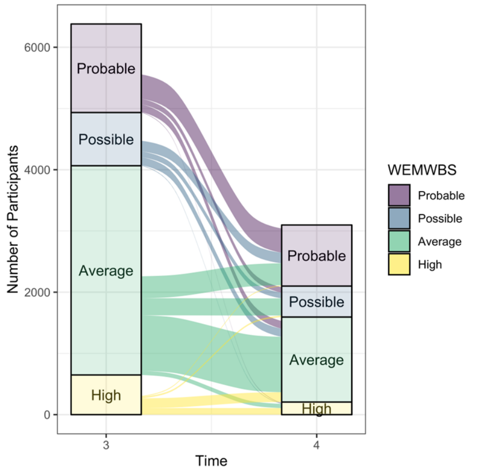

WEMWBS	Number (%) T3	T4
Probable MH Difficulties	1446 (22.7%)	997 (32.2%)
Possible MH Difficulties	869 (13.6%)	506 (16.3%)
Average MH	3417 (53.6%)	1388 (44.8%)
High Wellbeing	648 (10.2%)	206 (6.7%)

© 2023 Montero-Marin J et al. *JAMA Netw Open.*

eTable 4. Univariable analyses for risk for depression, social-emotional-behavioral difficulties, and well-being (Cohort 1)

	CES-D				SDQ				WEMWBS			
				LRT				LRT				LRT
	B	95% CI	p	p	B	95% CI	p	p	B	95% CI	p	p
Student characteristics												
Age	0.67	-0.73, 2.07	0.35	**<0.001**	0.29	-0.51, 1.10	0.47	**<0.001**	-0.14	-1.23, 0.96	0.81	**0.02**
*Time*Age*	-0.28	-1.53, 0.97	0.66		0.06	-0.56, 0.68	0.85		0.17	-0.93, 1.28	0.76	
Gender female (vs male)	4.30	2.69, 5.91	**<0.001**	**0.006**	0.94	0.002, 1.88	**0.05**	0.65	-2.36	-3.63, -1.09	**<0.001**	0.89
Gender Other/Prefer not to say (vs male)	9.25	4.08, 14.42	**<0.001**		3.06	0.05, 6.07	**0.05**		-6.16	-10.25, -2.08	**0.003**	
*Time*Female*	2.09	0.71, 3.47	**0.003**			NA				NA		
*Time*Other/Prefer not to say*	4.16	-0.49, 8.81	0.08			NA				NA		
Ethnicity white (vs other ethnic groups)	0.70	-1.33, 2.74	0.50	0.46	0.85	-0.31, 2.01	0.15	0.36	-0.60	-2.19, 0.99	0.46	0.35
*Time*Ethnicity white*		NA				NA				NA		
Year group 10 (vs Year group 9)	0.67	-0.99, 2.33	0.43	0.68	0.58	-0.37, 1.52	0.23	0.11	-0.12	-1.41, 1.17	0.85	0.82
*Time*Year group 10*		NA				NA				NA		
Student-rated school climate (student level)	-6.62	-7.62, -5.62	**<0.001**	**0.002**	-4.04	-4.60, -3.48	**<0.001**	**0.004**	5.21	4.44, 5.98	**<0.001**	0.91
*Time*Student-rated school climate*	1.54	0.56, 2.52	**0.002**		0.72	0.23, 1.20	**0.004**			NA		
Low risk for mental health difficulties (vs high)	-8.81	-10.48, -7.13	**<0.001**	**0.002**	-5.58	-6.52, -4.64	**<0.001**	**0.04**	6.34	5.02, 7.66	**<0.001**	**0.008**
*Time*Low risk for mental health difficulties*	2.47	0.90, 4.04	**0.002**		0.99	0.21, 1.77	**0.01**		-1.91	-3.30, -0.51	**0.007**	
Operational features of the school												
School size	0.01	0.002, 0.02	**0.009**	0.11	0.003	-0.001, 0.01	0.10	0.79	-0.01	-0.01, -0.001	**0.02**	0.33
*Time*School size*		NA				NA				NA		
Student to teacher ratio	0.49	-0.35, 1.34	0.25	0.23	0.10	-0.38, 0.58	0.67	0.87	-0.38	-1.01, 0.25	0.23	0.18
*Time*Student to teacher ratio*		NA				NA				NA		
Coeducational (vs female-only)	-0.81	-3.95, 2.33	0.61	**0.02**	0.14	-1.60, 1.89	0.87	0.47	0.71	-1.54, 2.97	0.53	**0.02**
*Time*Coeducational*	-2.50	-4.60, -0.39	**0.002**			NA			2.23	0.36, 4.09	**0.02**	
School quality, good (vs outstanding)	1.85	-0.52, 4.22	0.13	0.72	0.18	-1.28, 1.64	0.81	0.42	-1.85	-3.70, 0.00	**0.05**	0.49
School quality, req. Improv.	1.21	-2.26, 4.69	0.49		-0.55	-2.68, 1.58	0.61		-2.22	-4.93, 0.50	0.11	
*Time*School quality, good*		NA				NA				NA		
*Time*School quality, req. improv.*		NA				NA				NA		
SEL quality rating	0.09	-0.26, 0.44	0.61	0.07	0.12	-0.06, 0.31	0.20	0.24	-0.07	-0.33, 0.18	0.58	0.13
*Time*SEL quality rating*		NA				NA				NA		
SEL ethos	-0.02	-0.12, 0.08	0.67	0.22	0.02	-0.03, 0.08	0.43	0.08	0.03	-0.04, 0.10	0.46	0.21
*Time*SEL ethos*		NA				NA				NA		
School attainment	-0.18	-0.40, 0.04	0.12	0.51	-0.04	-0.18, 0.09	0.53	0.17	0.16	-0.01, 0.32	0.07	0.17
*Time*School attainment*		NA				NA				NA		
Teacher-rated school climate (sch. Level)	-4.30	-7.72, -0.88	**0.014**	0.52	-2.40	-4.36, -0.43	**0.02**	0.77	2.36	-0.32, 5.05	0.08	0.63
*Time*Teacher-rated school climate*		NA				NA				NA		
Student-rated school climate (sch. Level)	-9.65	-17.13, -2.18	**0.011**	0.52	-4.32	-8.61, -0.03	**0.05**	0.65	7.77	1.92, 13.61	**0.009**	0.78
*Time*Student-rated school climate*		NA				NA				NA		
Characteristics of the School community												
% Free school meals	0.16	-0.02, 0.34	0.08	0.75	0.09	-0.01, 0.19	0.07	0.93	-0.09	-0.22, 0.05	0.22	0.60
Time% Free school meals*		NA				NA				NA		
% SEND support	-0.06	-0.39, 0.27	0.73	0.79	-0.03	-0.21, 0.16	0.77	0.90	-0.04	-0.28, 0.20	0.75	0.30
Time% SEND support*		NA				NA				NA		
Students age (sch. Level)	-0.10	-4.35, 4.15	0.96	**<0.001**	-0.05	-2.42, 2.32	0.97	**<0.001**	-0.05	-3.14, 3.04	0.98	**0.02**
*Time*Students age*	0.65	-1.72, 3.02	0.59		-0.66	-1.83, 0.52	0.27		-0.90	-3.00, 1.21	0.40	
% Students white	0.04	-0.05, 0.13	0.08	0.46	0.02	-0.03, 0.07	0.44	0.51	-0.05	-0.11, 0.01	0.12	0.77
Time% Students white*		NA				NA				NA		
Broader context												
Urbanicity rural (vs urban)	-1.92	-4.57, 0.73	0.16	0.55	-1.91	-3.32, -0.50	**0.008**	**0.04**	0.32	-1.73, 2.37	0.76	0.60
*Time*Urbanicity rural*		NA			0.93	0.04, 1.81	**0.04**			NA		
Area level deprivation IMD	-0.53	-0.97, -0.09	**0.02**	0.78	-0.30	-0.58, -0.02	**0.04**	.92	0.33	-0.06, 0.72	0.09	0.71
*Time*Area level deprivation IMD*		NA				NA				NA		

Univariable analyses using multilevel linear regressions via Maximum Likelihood estimation and three-level mixed effects models for the analysis of the associations between student- and school-level characteristics and changes in adolescents' mental health and well-being between T3 and T4 in Cohort 1 (pre-pandemic). The first step includes the univariable analyses (e.g., age), and the second step includes the univariable analyses + the corresponding two-way interaction (e.g., *Time*Age*). LRT: Likelihood-ratio test comparing Step 1 vs Step 2. The continuous student-level factors (age, school climate) were group mean (school-level) centred, and therefore the regression coefficients represent an estimate of the differences in individual effects within schools. The continuous school-level factors were introduced as group means, so that these regression coefficients represent school-level effects (i.e., differences between schools).[29] Regression coefficients of the interaction terms reflect changes relative to the first assessment (i.e., T4 vs. T3). All models controlled for design variables, trial arm allocation, and the time difference (days) between T3-T4. The 'other ethnic groups' category includes Arab, Asian, Black/African/Caribbean, mixed/multiple ethnic groups, other ethnic groups. CES-D: Center for Epidemiologic Studies for Depression Scale (range: 0-60). SDQ: Strengths and Difficulties Questionnaire (Total Difficulties Score; range: 0-40). WEMWBS: Warwick-Edinburgh Mental Well-Being Scale (range: 14-70). NA: not applicable given the absence of significant results in the LRT comparing Step 1 vs Step 2.

© 2023 Montero-Marin J et al. *JAMA Netw Open.*

eTable 5. Univariable analyses of risk for depression, social-emotional-behavioral difficulties, and well-being (Cohort 2)

	CES-D				SDQ				WEMWBS			
				LRT				LRT				LRT
	B	95% CI	p	p	B	95% CI	p	p	B	95% CI	p	p
Student characteristics												
Age	0.95	0.28, 1.62	**0.005**	**0.04**	0.22	-0.15, 0.60	0.24	0.06	-0.47	-1.00, 0.05	0.08	**0.02**
*Time*Age*	1.30	0.55, 2.04	**0.001**			NA			0.68	0.10, 1.25	**0.02**	
Gender female (vs male)	7.02	6.17, 7.86	**<0.001**	**<0.001**	2.75	2.26, 3.23	**<0.001**	**<0.001**	-4.87	-5.54, -4.20	**<0.001**	**<0.001**
Gender Other/Prefer not to say (vs male)	7.59	4.80, 10.37	**<0.001**		2.98	1.38, 4.57	**<0.001**		-5.44	-7.65, -3.23	**<0.001**	
*Time*Female*	2.10	1.28, 2.92	**<0.001**		1.39	0.98, 1.81	**<0.001**		-1.66	-2.35, -0.97	**<0.001**	
*Time*Other/Prefer not to say*	0.92	-2.06, 3.90	0.55		0.27	-1.22, 1.77	0.72		-2.42	-4.92, 0.09	0.06	
Ethnicity white (vs other ethnic groups)	0.41	-0.58, 1.40	0.42	0.94	0.77	0.23, 1.32	**0.006**	0.49	-0.55	-1.32, 0.23	0.17	0.10
*Time*Ethnicity white*		NA				NA				NA		
Year group 10 (vs Year group 9)	1.43	0.55, 2.31	**0.001**	0.96	0.65	0.16, 1.13	**0.009**	**0.04**	-1.13	-1.82, -0.45	**0.001**	0.05
*Time*Year group 10*		NA			-0.42	-0.83, -0.02	**0.04**			NA		
Student-rated school climate (student level)	-5.67	-6.19, -5.16	**<0.001**	**<0.001**	-3.38	-367, -3.10	**<0.001**	**<0.001**	4.59	4.19, 4.99	**<0.001**	**<0.001**
*Time*Student-rated school climate*	1.52	0.95, 2.09	**<0.001**		1.08	0.80, 1.37	**<0.001**		-1.38	-1.86, -0.90	**<0.001**	
Low risk for mental health difficulties (vs high)	-9.20	-10.04, -8.37	**<0.001**	**<0.001**	-5.51	-5.97, -5.05	**<0.001**	**<0.001**	6.69	6.03, 7.36	**<0.001**	**0.001**
*Time*Low risk for mental health difficulties*	1.94	1.02, 2.86	**<0.001**		1.18	0.72, 1.65	**<0.001**		-1.31	-2.08, -0.53	**0.001**	
Home environment												
Household assets	-6.32	-8.39, -4.26	**<0.001**	**0.02**	-3.57	-4.72, -2.42	**<0.001**	0.12	5.10	3.51, 6.70	**<0.001**	0.61
*Time*Household assets*	2.36	0.42, 4.30	**0.02**			NA				NA		
Studying conditions	-18.27	-20.51, -16.04	**<0.001**	**0.003**	-10.06	-11.31, -8.80	**<0.001**	0.11	12.77	11.04, 14.50	**<0.001**	**0.02**
*Time*Studying conditions*	-3.61	-5.98, -1.23	**0.003**			NA			2.30	0.31, 4.28	**0.02**	
Home connectedness	-23.93	-25.29, -22.57	**<0.001**	**<0.001**	-12.19	-12.98, -11.39	**<0.001**	**<0.001**	18.10	17.04, 19.17	**<0.001**	**<0.001**
*Time*Home connectedness*	-7.23	-8.86, -5.60	**<0.001**		-2.03	-2.84, -1.21	**<0.001**		4.88	3.52, 6.24	**<0.001**	
Home conflicts sometimes (vs lots of times)	-3.55	-4.82, -2.29	**<0.001**	**<0.001**	-1.65	-2.37, -0.94	**<0.001**	**0.001**	1.75	0.76, 2.75	**0.001**	**0.01**
Home conflicts rarely	-6.42	-7.64, -5.20	**<0.001**		-3.31	-4.00, -2.63	**<0.001**		4.16	3.20, 5.11	**<0.001**	
Home conflicts no/don't know	-9.28	-10.41, -8.15	**<0.001**		-4.74	-5.37, -4.10	**<0.001**		5.99	5.11, 6.88	**<0.001**	
*Time*Home conflicts sometimes*	-0.69	-2.03, 0.65	0.31		-0.38	-1.05, 0.30	0.27		0.50	-0.63, 1.63	0.39	
*Time*Home conflicts rarely*	-1.83	-3.12, -0.54	**0.006**		-0.69	-1.34, -0.04	**0.04**		1.18	0.09, 2.26	**0.03**	
*Time*Home conflicts no/don't know*	-3.23	-4.42, -2.05	**<0.001**		-1.13	-1.73, -0.53	**<0.001**		1.57	0.57, 2.58	**0.002**	
Friendships												
Friend, don't know (vs yes)	6.84	5.25, 8.43	**<0.001**	**0.007**	3.38	2.49, 4.28	**<0.001**	**0.04**	-5.26	-6.49, -4.04	**<0.001**	0.16
Friend, no (vs yes)	6.99	5.40, 8.58	**<0.001**		4.20	3.31, 5.09	**<0.001**		-5.98	-7.21, -4.75	**<0.001**	
Friend, prefer not to say (vs yes)	7.51	4.65, 10.37	**<0.001**		4.18	2.57, 5.78	**<0.001**		-5.53	-7.74, -3.32	**<0.001**	
*Time*Friend, don't know*	2.93	1.27, 4.59	**0.001**		0.93	0.10, 1.76	**0.03**			NA		
*Time*Friend, no*	0.59	-1.07, 2.26	0.48		-0.11	-0.93, 0.72	0.80			NA		
*Time*Friend, prefer not to say*	0.001	-3.00, 3.00	0.99		1.48	-0.01, 2.97	0.06			NA		
Operational features of the school												
School size	-0.001	-0.004, 0.02	0.45	**0.02**	0.0002	-0.002, 0.002	0.85	0.21	0.0003	-0.002, 0.003	0.84	0.79
*Time*School size*	0.002	0.0003, 0.003	**0.02**			NA				NA		
Student to teacher ratio	0.10	-0.29, 0.49	0.62	0.86	-0.03	-0.24, 0.18	0.79	0.17	-0.10	-0.39, 0.19	0.50	0.36
*Time*Student to teacher ratio*		NA				NA				NA		
Coeducational (vs female-only)	-2.60	-4.09, -1.11	**0.001**	0.22	-0.67	-1.46, -0.11	0.09	**0.006**	1.78	0.63, 2.94	**0.002**	**0.02**
*Time*Coeducational*		NA			-0.66	-1.12, -0.19	**0.006**		0.94	0.15, 1.72	**0.02**	
School quality, good (vs outstanding)	0.93	-1.05, 2.91	0.36	**0.04**	0.65	-0.41, 1.71	0.23	0.22	-0.73	-2.23, 0.78	0.34	0.83
School quality, req. Improv.	1.40	-1.78, 4.59	0.39		0.58	-1.14, 2.29	0.51		-1.87	-4.30, 0.56	0.13	
*Time*School quality, good*	-0.09	-1.01, 0.83	0.84			NA				NA		
*Time*School quality, req. improv.*	-2.33	-4.18, -0.49	**0.01**			NA				NA		
SEL quality rating	-0.20	-0.52, 0.11	0.20	0.62	-0.17	-0.33, -0.01	**0.04**	0.43	0.13	-0.11, 0.38	0.28	**0.007**
*Time*SEL quality rating*		NA				NA			0.21	0.06, 0.37	**0.007**	
SEL ethos	-0.06	-0.13, 0.02	0.15	0.06	-0.03	-0.07, 0.01	0.13	0.34	0.05	-0.01, 0.10	0.12	0.23
*Time*SEL ethos*		NA				NA				NA		
School attainment	-0.03	-0.07, 0.02	0.22	0.39	-0.01	-0.03, 0.01	0.35	0.67	0.01	-0.02, 0.04	0.57	0.96
*Time*School attainment*		NA				NA				NA		
School (during lockdown) some of the time (vs at home)	2.84	1.20, 4.48	**0.001**	0.78	2.26	1.34, 3.17	**<0.001**	0.86	-1.61	-2.88, -0.34	**0.01**	0.33
School (during lockdown) full time	0.20	-1.26, 1.66	0.79		0.49	-0.33, 1.31	0.24		0.41	-0.72, 1.54	0.48	
*Time*School some of the time*		NA				NA				NA		
*Time*School full time*		NA				NA				NA		
Teacher-rated school climate (sch. Level)	-0.75	-2.89, 1.38	0.49	0.24	-0.003	-1.13, 1.12	0.99	0.87	0.01	-1.65, 1.67	0.99	0.88
*Time*Teacher-rated school climate*		NA				NA				NA		
Student-rated school climate (sch. Level)	-6.10	-9.38, -2.82	**<0.001**	.41	-2.40	-4.22, -0.58	**0.01**	0.82	4.19	1.54, 6.84	**0.002**	.21
*Time*Student-rated school climate*		NA				NA				NA		
Characteristics of the school community												
% Free school meals (school deprivation)	0.08	-0.02, 0.18	0.12	0.39	0.03	-0.02, 0.08	0.29	0.08	-0.06	-0.14, 0.02	0.15	0.80
Time% Free school meals*		NA				NA				NA		
% SEND support	0.09	-0.05, 0.23	0.22	0.22	0.07	0.003, 0.14	**0.04**	0.27	-0.08	-0.19, 0.03	0.14	0.56
Time% SEND support*		NA				NA				NA		
Students age (sch. Level)	1.26	-1.19, 3.71	0.32	**<0.001**	0.58	-0.71, 1.87	0.38	**<0.001**	-1.07	-2.96, 0.83	0.27	**<0.001**
*Time*Students age*	1.76	0.24, 3.29	**0.02**		0.02	-0.76, 0.79	0.97		0.21	-1.09, 1.50	0.76	
% Students white	0.004	-0.02, 0.03	0.78	0.81	0.01	-0.01, 0.02	0.30	0.40	0.001	-0.02, 0.02	0.95	**0.04**
Time% Students white*		NA				NA			0.01	0.001, 0.03	**0.04**	
Broader context												
Urbanicity rural (vs urban)	-1.38	-3.20, 0.45	0.14	0.72	-0.44	-1.41, 0.53	0.37	0.67	1.70	0.35, 3.06	**0.01**	0.74
*Time*Urbanicity rural*		NA				NA				NA		
Area level deprivation IMD	-0.20	-0.45, 0.05	0.12	0.58	-0.09	-0.22, -0.04	0.19	0.09	0.21	0.02, 0.40	**0.03**	0.46
*Time*Area level deprivation IMD*		NA				NA				NA		

Univariable analyses using multilevel linear regressions via Maximum Likelihood estimation and three-level mixed effects models for the analysis of the associations between student- and school-level characteristics and changes in adolescents' mental health and well-being between T3 and T4 in Cohort 2 (pandemic). The first step includes the univariable analyses (e.g., age), and the second step includes the univariable analyses + the corresponding two-way interaction (e.g., *Time*Age*). LRT: Likelihood-ratio test comparing Step 1 vs Step 2. The continuous student-level factors (age, school climate) were group mean (school-level) centred, and therefore the regression coefficients represent an estimate of the differences in individual effects within schools. The continuous school-level factors were introduced as group means, so that these regression coefficients represent school-level effects (i.e., differences between schools).[29] Regression coefficients of the interaction terms reflect changes relative to the first assessment (i.e., T4 vs. T3). All models controlled for design variables, trial arm allocation, and the time difference (days) between T3 and T4. The 'other ethnic groups' category includes Arab, Asian, Black/African/Caribbean, mixed/multiple ethnic groups, other ethnic groups. CES-D: Center for Epidemiologic Studies for Depression Scale (range: 0-60). SDQ: Strengths and Difficulties Questionnaire (Total Difficulties Score; range: 0-40). WEMWBS: Warwick-Edinburgh Mental Well-Being Scale (range: 14-70). NA: not applicable given the absence of significant results in the LRT comparing Step 1 vs Step 2.

© 2023 Montero-Marin J et al. *JAMA Netw Open.*

eTable 6. Multivariable analyses of risk for depression, social-emotional-behavioral difficulties, and well-being (Cohort 1)

	CES-D			SDQ			WEMWBS		
	B	95% CI	p	B	95% CI	p	B	95% CI	p
Student characteristics									
Gender female (vs male)	3.77	2.20, 5.34	**<0.001**	0.89	0.11, 1.67	**0.03**†	-2.57	-3.67, -1.47	**<0.001**
Gender Other/Prefer not to say (vs male)	7.84	3.40, 12.28	**0.001**	2.07	-0.47, 4.60	0.11	-5.54	-9.02, -2.06	**0.002**
*Time*Female*	1.43	-0.01, 2.87	0.06		NA			NA	
Student-rated school climate (student level)	-6.52	-7.59, -5.44	**<0.001**	-3.85	-4.45, -3.25	**<0.001**	4.67	3.93, 5.42	**<0.001**
*Time*Student-rated school climate*	1.30	0.30, 2.30	**0.01**	0.66	0.16, 1.15	**0.009**		NA	
Low risk for mental health difficulties (vs high)	-7.43	-9.21, -5.66	**<0.001**	-4.44	-5.43, -3.44	**<0.001**	5.73	4.31, 7.16	**<0.001**
*Time*Low risk for mental health difficulties*	2.01	0.37, 3.64	**0.02**	0.54	-0.27, 1.34	0.20	-2.13	-3.56, -0.70	**0.003**
Operational features of the school									
School size	0.002	-0.004, 0.01	0.48		NA		-0.01	-0.01, 0.0001	0.06
Coeducational (vs female-only)	3.72	0.84, 6.60	**0.01**		NA		-1.88	-4.14, 0.39	0.10
*Time*Coeducational*	-1.91	-4.11, 0.30	0.09		NA		2.44	0.57, 4.31	**0.01**
School quality, good (vs outstanding)		NA			NA		-0.31	-1.58, 0.96	0.63
Teacher-rated school climate (sch. Level)	-1.73	-6.33, 2.88	0.46	-2.27	-5.37, 0.82	0.15		NA	
Student-rated school climate (sch. Level)	-3.24	-14.43, 7.94	0.57	0.79	-5.73, 7.31	0.81		NA	
Broader context									
Urbanicity rural (vs urban)		NA		-0.53	-2.27, 1.20	0.546		NA	
*Time*Urbanicity rural*		NA		0.88	-0.01, 1.77	0.052		NA	
Area level deprivation IMD	-0.46	-0.95, 0.03	0.068	-0.12	-0.39, 0.16	0.416		NA	

Multivariable analyses, using multilevel linear regressions via Maximum Likelihood estimation and three-level mixed effects models for the analysis of the unique associations between student- and school-level characteristics and changes in adolescents' mental health and well-being between T3 and T4 in Cohort 1 (pre-pandemic), entering those factors that provided significant p-values ($p < 0.05$) in the previous univariable analyses (eTable 4). The continuous student-level factors (school climate) were group mean (school-level) centred, and therefore the regression coefficients represent an estimate of the differences in individual effects within schools. The continuous school-level factors were introduced as group means, so that these regression coefficients represent school-level effects (i.e., differences between schools).[29] Regression coefficients of the interaction terms reflect changes relative to the first assessment (i.e., T4 vs. T3). All models controlled for design variables, trial arm allocation, and the time difference (days) between T3 and T4. CES-D: Center for Epidemiologic Studies for Depression Scale (range: 0-60). SDQ: Strengths and Difficulties Questionnaire (Total Difficulties Score; range: 0-40). WEMWBS: Warwick-Edinburgh Mental Well-Being Scale (range: 14-70). † This relationship was no longer significant when the Benjamini-Hochberg correction was applied to correct for multiple testing. NA: Not applicable given the absence of significant results in the univariable analysis. Those variables that did not show significant univariable associations in any of the outcome variables are omitted.

© 2023 Montero-Marin J et al. *JAMA Netw Open.*

eFigure 3. Relationship between student-rated school climate and outcomes as a function of time (Cohort 1)

In the pre-pandemic Cohort 1, a more positive student-rated school climate (at T3) was related to a decreasing risk for depression and social-emotional-behavioral difficulties, as well as improved mental well-being, but the association between school climate and risk for depression and social-emotional-behavioral difficulties weakened over time (B=1.30 (95%CI=0.30, 2.30), B=0.66 (95%CI=0.16, 1.15), respectively) (see eTable 6).

3.1: Risk for Depression: Center for Epidemiological Studies for Depression Scale (CESD)

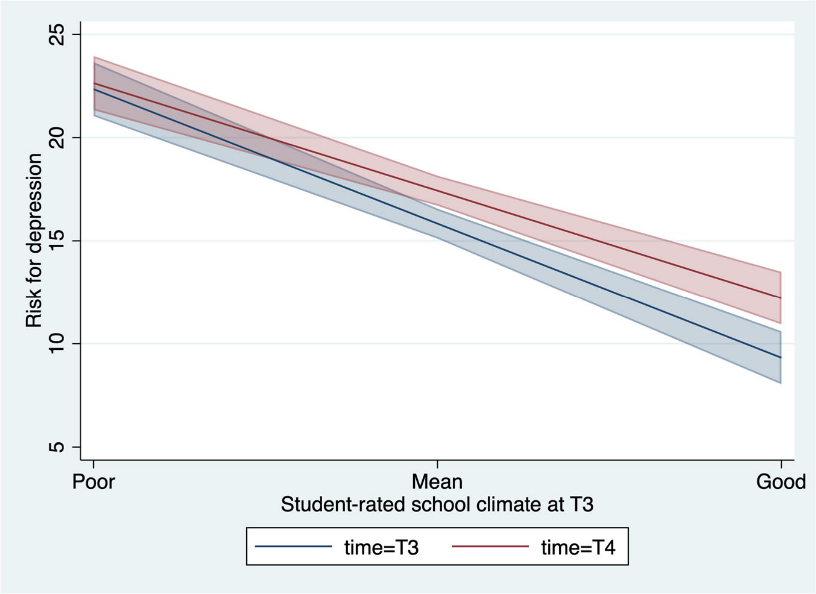

Risk for depression scores are predictive margins (eTable 6).

3.2: Social-emotional-behavioral difficulties: Strengths and Difficulties Questionnaire (SDQ)

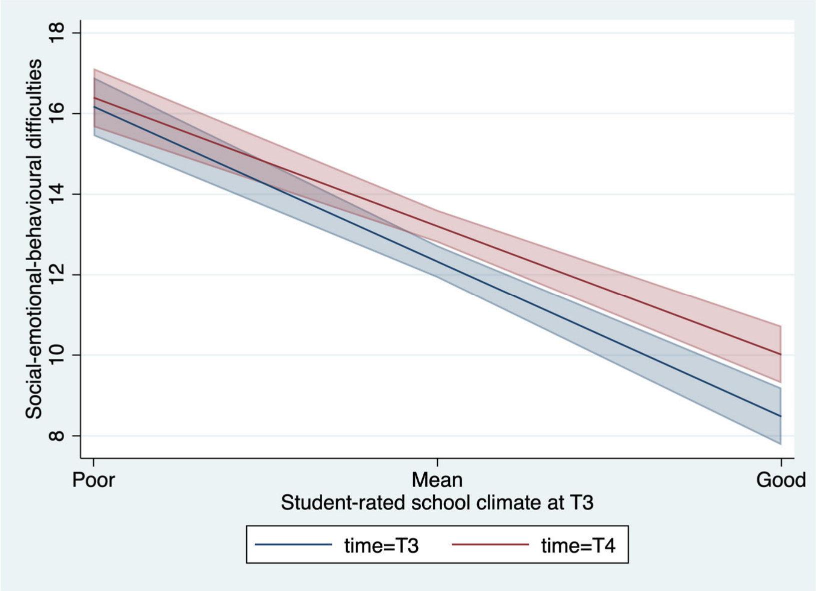

Social-emotional-behavioral difficulties scores are predictive margins (eTable 6).

© 2023 Montero-Marin J et al. *JAMA Netw Open.*

eFigure 4. Relationship between coeducation and outcomes as a function of time (Cohort 1)

In the pre-pandemic Cohort 1, students in a coeducational school were associated with lower decreases in mental well-being between T3 and T4 (B=2.44, 95% CI=0.57 to 4.31) compared to those in a female-only school, who experienced higher decreases in mental well-being between T3 and T4 (see eTable 6).

4.1: Mental well-being: Warwick-Edinburgh Mental Well-being Scale (WEMWBS)

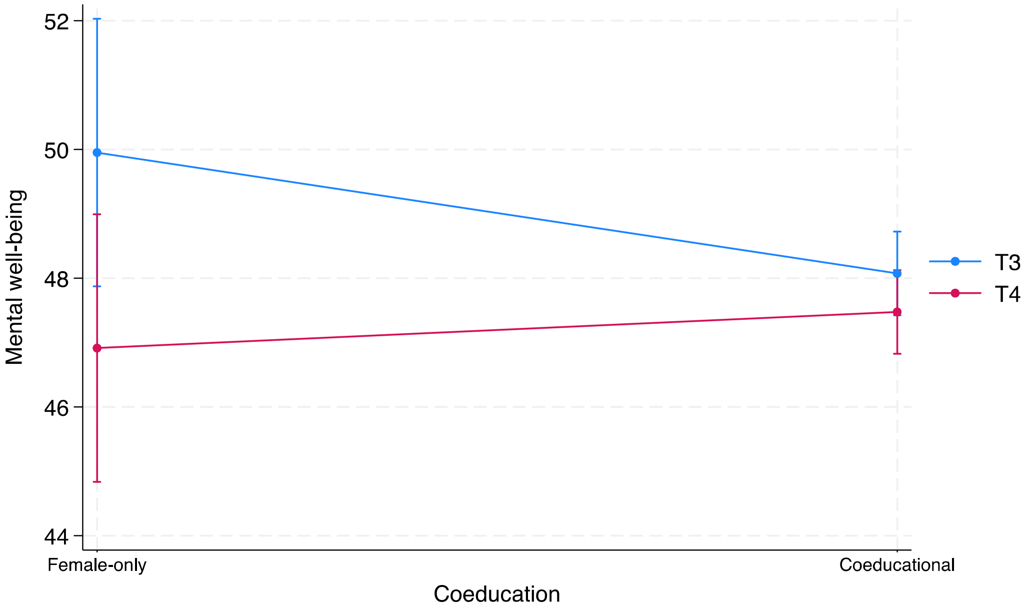

Mental well-being scores (possible range: 14 to 70) are predictive margins (eTable 6).

© 2023 Montero-Marin J et al. *JAMA Netw Open.*

eTable 7. Descriptive data and within-cohort outcome analyses by initial risk for mental health difficulties

	CESD T3	CESD T4				SDQ T3	SDQ T4				WEMWBS T3	WEMWBS T4			
	**M (SD)**	**M (SD)**	**AMD (95% CI)**	**p**	**d**	**M (SD)**	**M (SD)**	**AMD (95% CI)**	**p**	**d**	**M (SD)**	**M (SD)**	**AMD (95% CI)**	**p**	**d**
**Cohort 1** *(Pre-pandemic)*															
High risk	N=188					N=188					N=188				
	23.42 (11.85)	23.08 (12.25)	-0.34 (-1.74 to 1.06)	0.63	-0.04	16.90 (6.24)	17.02 (6.10)	0.12 (-0.60 to 0.83)	0.75	0.02	42.63 (9.34)	43.22 (9.05)	0.60 (-0.71 to 1.90)	0.37	0.06
Low risk	N=576					N=575					N=580				
	13.31 (10.46)	15.40 (11.36)	2.13 (1.36 to 2.91)	**<0.001**	0.22	10.83 (6.14)	11.93 (6.30)	1.11 (0.73 to 1.49)	**<0.001**	0.24	50.09 (8.80)	48.76 (9.10)	-1.31 (-1.99 to -0.64)	**<0.001**	-0.16
**Cohort 2** *(Pandemic)*															
High risk	N=693					N=682					N=696				
	24.63 (12.20)	26.62 (12.04)	1.95 (1.09 to 2.81)	**<0.001**	0.17	17.36 (6.29)	18.09 (5.98)	0.72 (0.30 to 1.13)	**0.001**	0.13	42.16 (8.74)	40.18 (9.18)	-1.92 (-2.61 to -1.23)	**<0.001**	-0.21
Low risk	N=2241					N=2221					N=2252				
	14.38 (10.37)	18.30 (11.90)	3.89 (3.46 to 4.33)	**<0.001**	0.37	11.26 (6.05)	13.16 (6.28)	1.90 (1.67 to 2.12)	**<0.001**	0.35	49.58 (8.94)	46.32 (9.53)	-3.23 (-3.60 to -2.85)	**<0.001**	-0.36

Mixed-effects linear regressions with maximum likelihood (ML) estimation, including schools (clusters) as random effects and adjusted for the country, school size, coeducation, allocation group and the time difference (days) between T3 and T4. Descriptives are raw data. M (SD): mean (standard deviation). AMD: adjusted mean difference. 95% CI: 95% confidence interval. P: p-value. D: Cohen's d effect size. CES-D: Center for Epidemiological Studies for Depression Scale (range: 0-60). SDQ: Strengths and Difficulties Questionnaire (Total Difficulties Score; range: 0-40). WEMWBS: Warwick-Edinburgh Mental Well-being Scale (range: 14-70). Significant differences remained significant when the Benjamini-Hochberg correction was applied to correct for multiple testing. See the eMethods for a description on how the initial risk of mental health difficulties status was estimated. In the pre-pandemic cohort 1, those with a low initial risk for mental health difficulties showed a deterioration in all outcomes over time. This deterioration was greater for those with a low initial risk compared to those with a high initial risk for mental health difficulties, for the outcomes risk for depression (B=2.01 (95% CI=0.37, 3.64) and mental well-being (B=-2.13 (95% CI=-3.56, -0.70) (see eTable 6). In the pandemic-exposed Cohort 2, both those with low and high initial risk for mental health difficulties showed deteriorations in outcomes. Deteriorations were greater in those with low (vs. high) initial risk for mental health difficulties (risk for depression (B=2.07 (95%CI=0.82, 3.32), social-emotional-behavioral difficulties (B=0.99 (95%CI=0.41, 1.57)) (see Table 3 (main manuscript)).

© 2023 Montero-Marin J et al. *JAMA Netw Open.*

eFigure 5. Outcomes by initial risk for mental health difficulties, cohort status, and time point

5.1: Risk for Depression

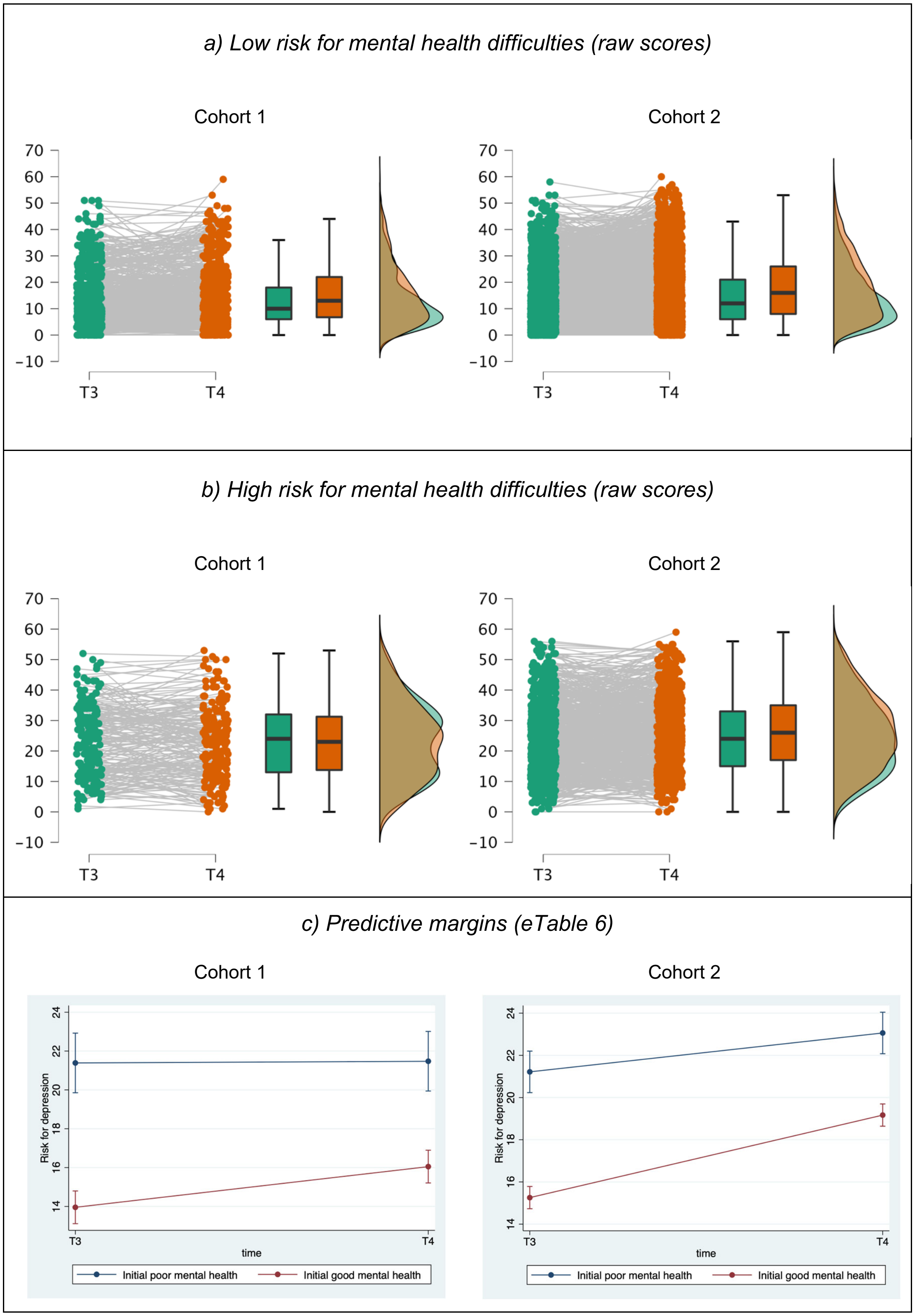

Risk for Depression: Center for Epidemiological Studies for Depression Scale (CES-D).

© 2023 Montero-Marin J et al. *JAMA Netw Open.*

5.2: Social-emotional-behavioral difficulties

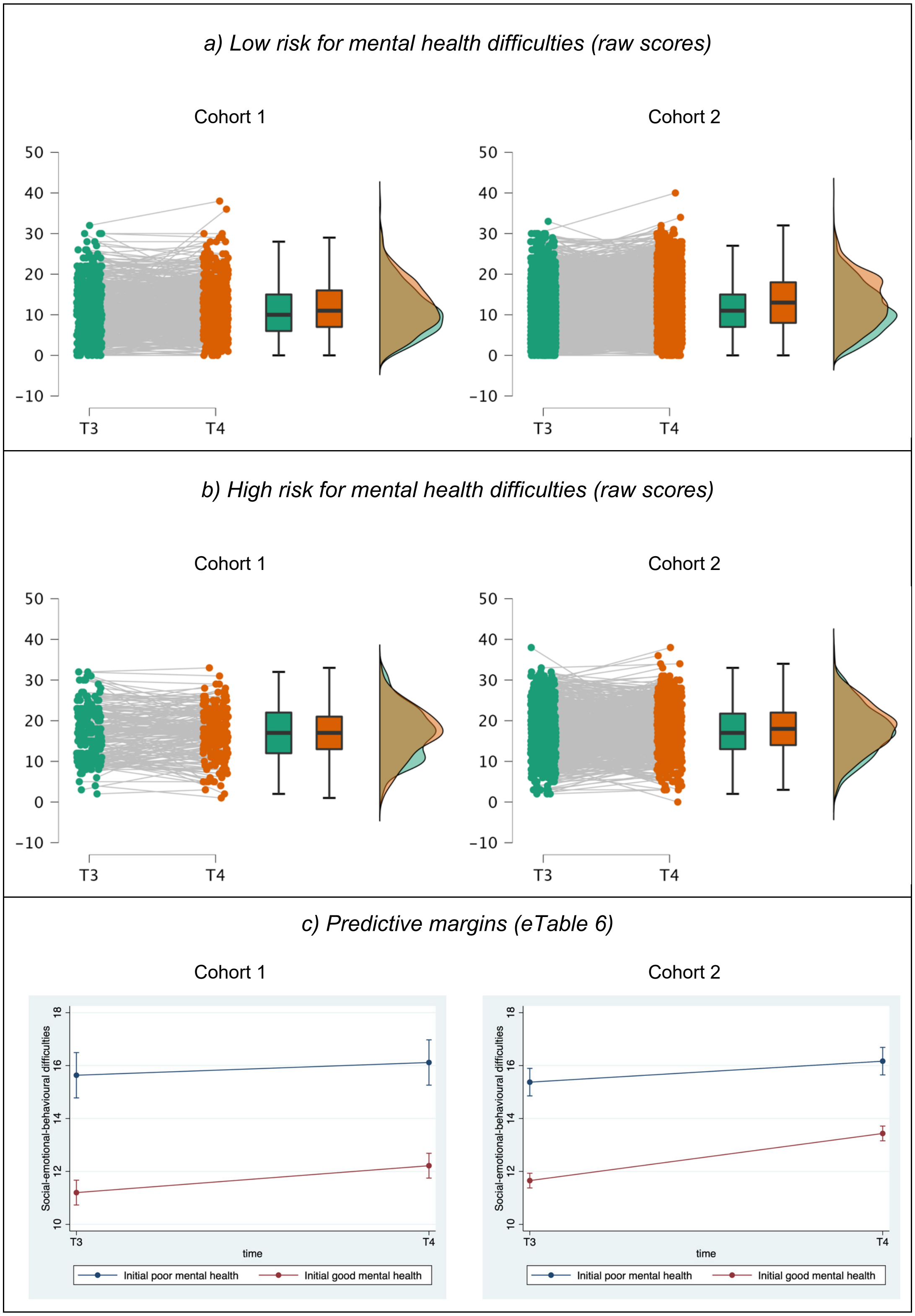

Social-emotional-behavioral difficulties: Strengths and Difficulties Questionnaire (SDQ).

© 2023 Montero-Marin J et al. *JAMA Netw Open.*

5.3: Mental well-being

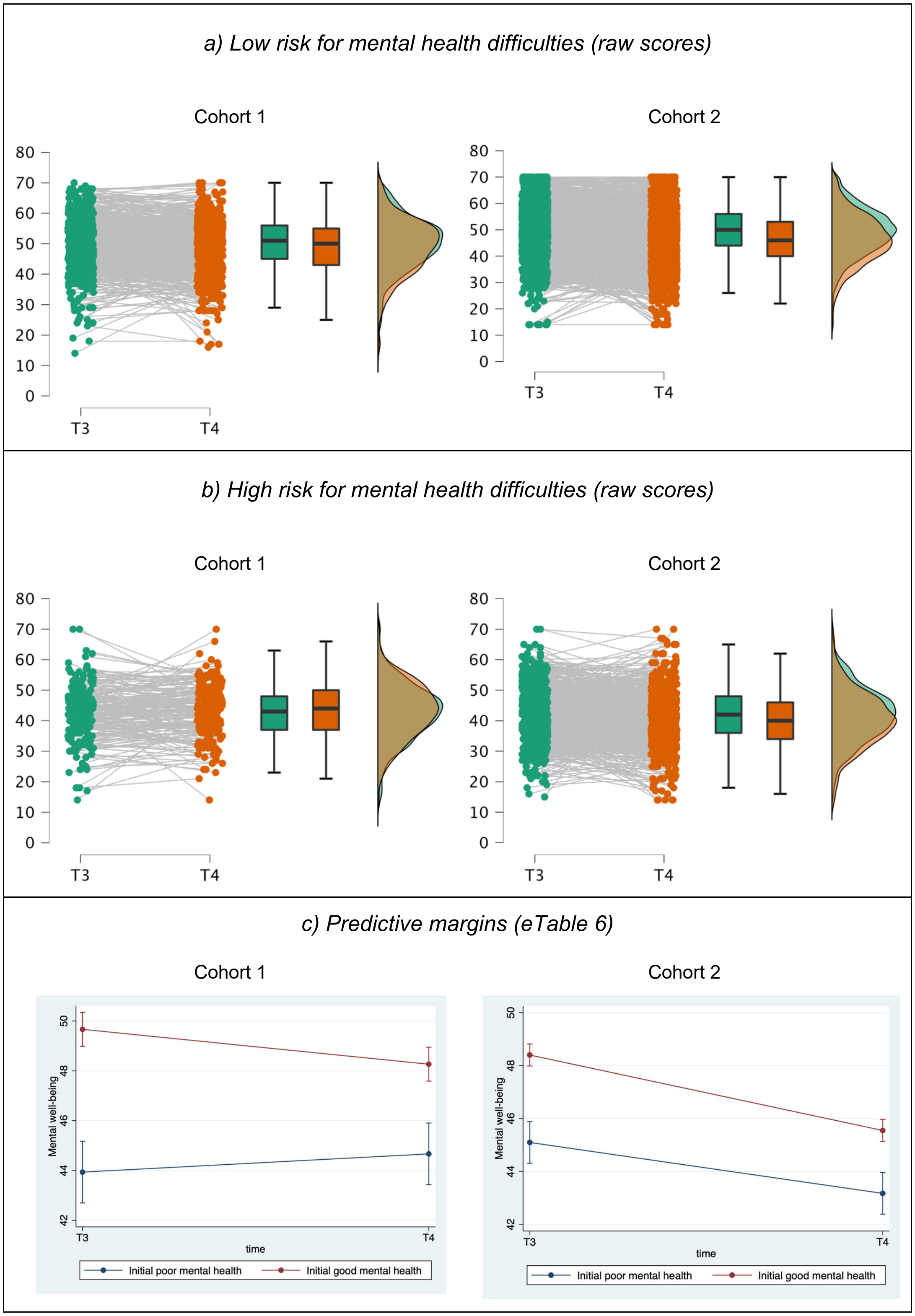

Mental well-being: Warwick-Edinburgh Mental Well-being Scale (WEMWBS).

© 2023 Montero-Marin J et al. *JAMA Netw Open.*

eFigure 6. Relationship between gender (female vs. male) and outcomes as a function of time (Cohort 2)

6.1: Risk for Depression: Center for Epidemiological Studies for Depression Scale (CESD)

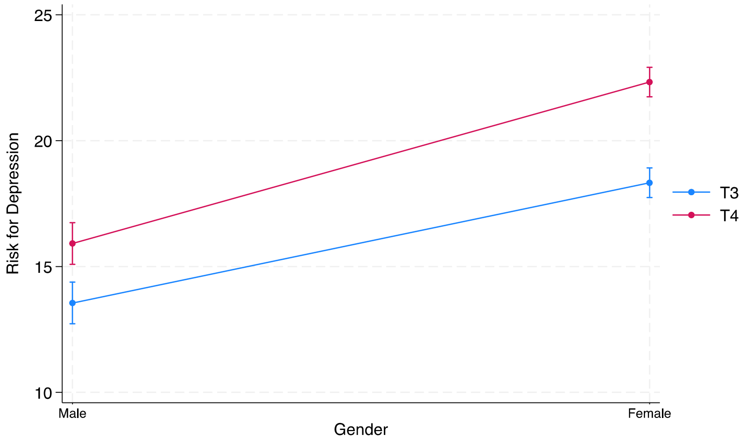

Risk for depression scores (possible range: 0 to 60) are predictive margins (Table 3).

6.2: Social-emotional-behavioral difficulties: Strengths and Difficulties Questionnaire (SDQ)

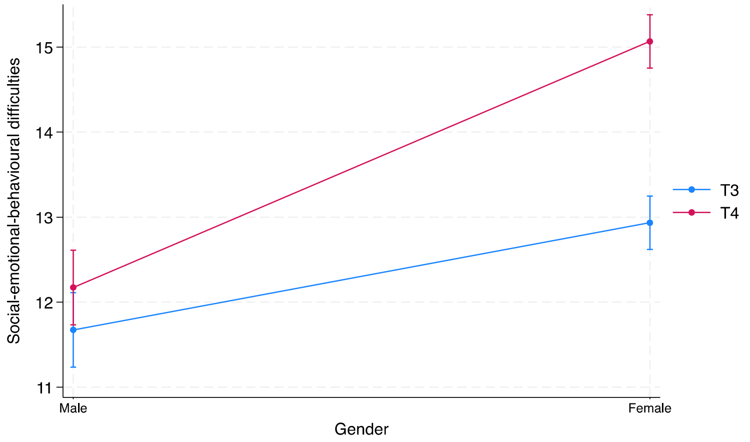

Social-emotional-behavioral difficulties scores (possible range: 0 to 40) are predictive margins (Table 3).

6.3: Mental well-being: Warwick-Edinburgh Mental Well-being Scale (WEMWBS)

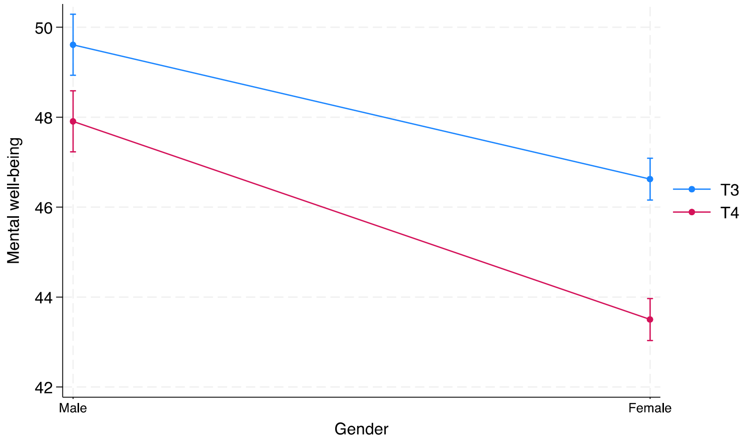

Mental well-being scores (possible range: 14 to 70) are predictive margins (Table 3).

© 2023 Montero-Marin J et al. *JAMA Netw Open.*

eFigure 7. Relationship between home connectedness and outcomes as a function of time (Cohort 2)

7.1: Risk for Depression: Center for Epidemiological Studies for Depression Scale (CESD)

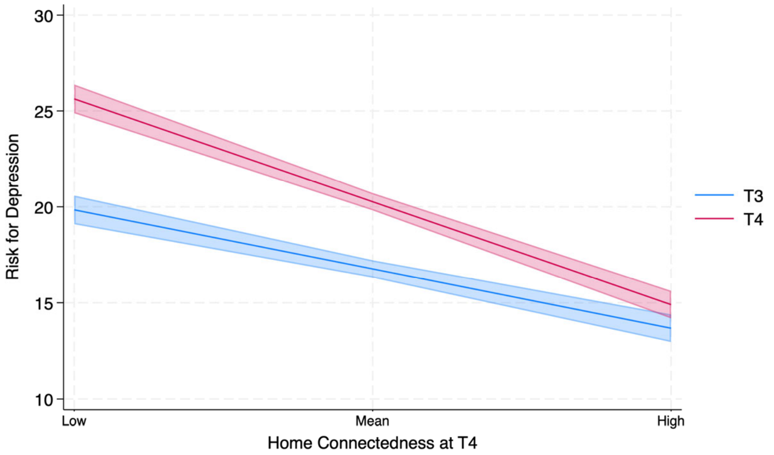

Risk for depression scores (possible range: 0 to 60) are predictive margins (Table 3).

7.2: Social-emotional-behavioral difficulties: Strengths and Difficulties Questionnaire (SDQ)

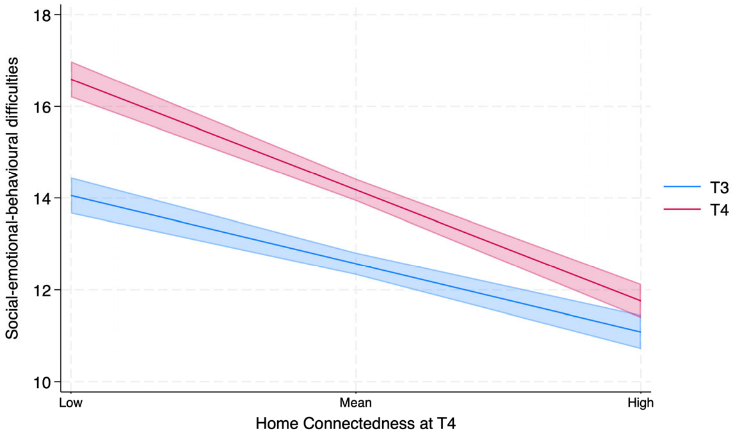

Social-emotional-behavioral difficulties scores (possible range: 0 to 40) are predictive margins (Table 3).

7.3: Mental well-being: Warwick-Edinburgh Mental Well-being Scale (WEMWBS)

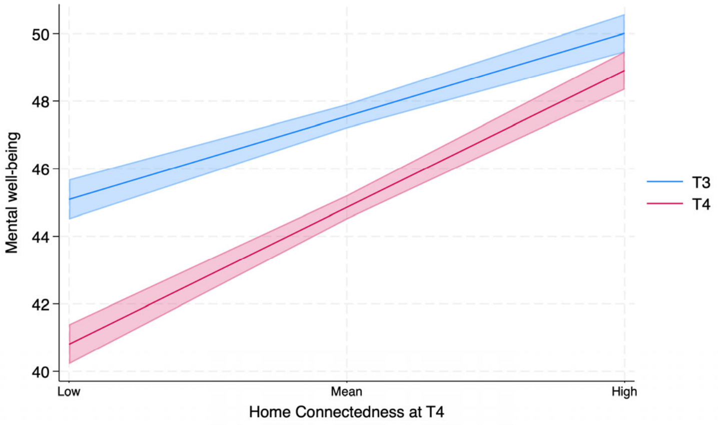

Mental well-being scores (possible range: 14 to 70) are predictive margins (Table 3).

© 2023 Montero-Marin J et al. *JAMA Netw Open.*

eFigure 8. Relationship between student-rated school climate and outcomes as a function of time (Cohort 2)

8.1: Risk for Depression: Center for Epidemiological Studies for Depression Scale (CESD)

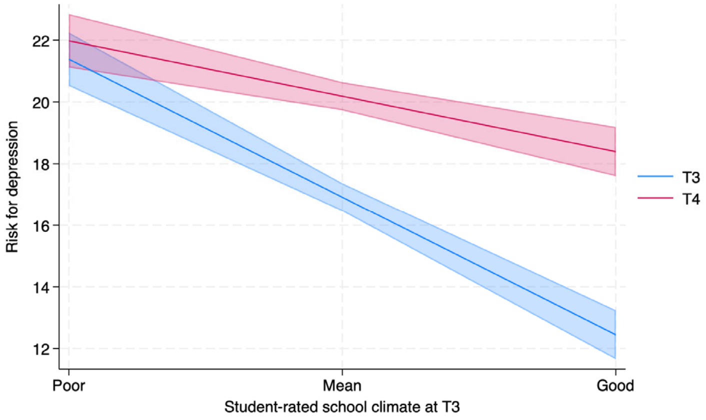

Risk for depression scores (possible range: 0 to 60) are predictive margins (Table 3).

8.2: Social-emotional-behavioral difficulties: Strengths and Difficulties Questionnaire (SDQ)

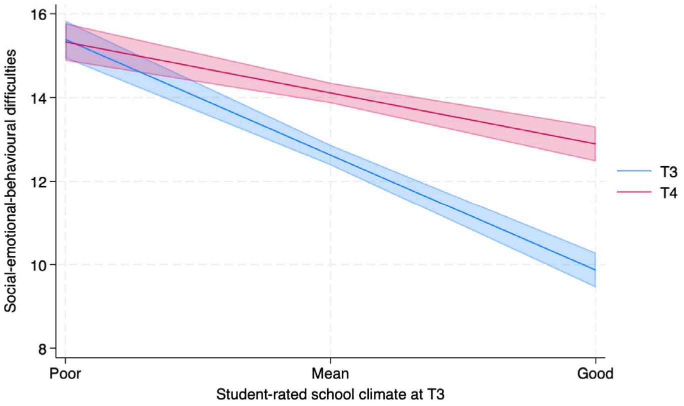

Social-emotional-behavioral difficulties scores (possible range: 0 to 40) are predictive margins (Table 3).

8.3: Mental well-being: Warwick-Edinburgh Mental Well-being Scale (WEMWBS)

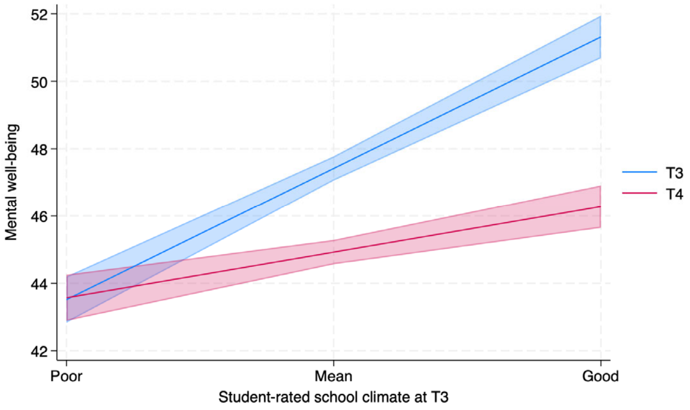

Mental well-being scores (possible range: 14 to 70) are predictive margins (Table 3).

© 2023 Montero-Marin J et al. *JAMA Netw Open.*

eFigure 9. Relationship between friendship and outcomes as a function of time (Cohort 2)

9.1: Risk for Depression: Center for Epidemiological Studies for Depression Scale (CESD)

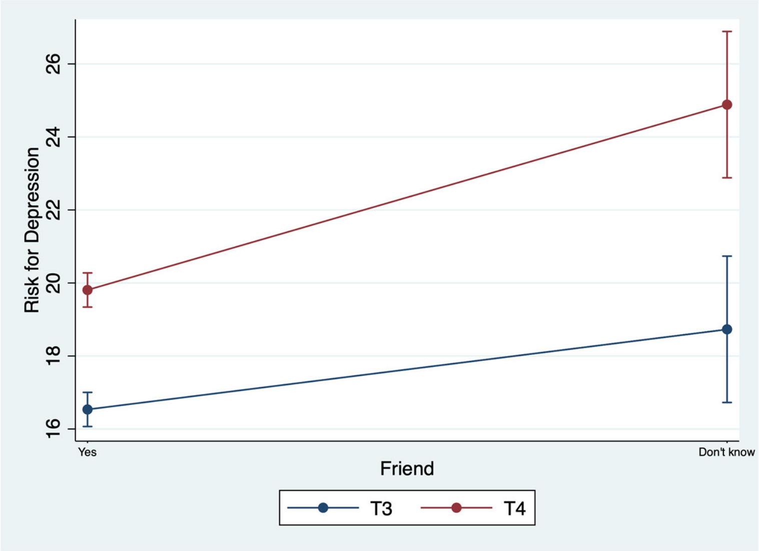

Risk for depression scores (possible range: 0 to 60) are predictive margins (Table 3).

9.2: Social-emotional-behavioral difficulties: Strengths and Difficulties Questionnaire (SDQ)

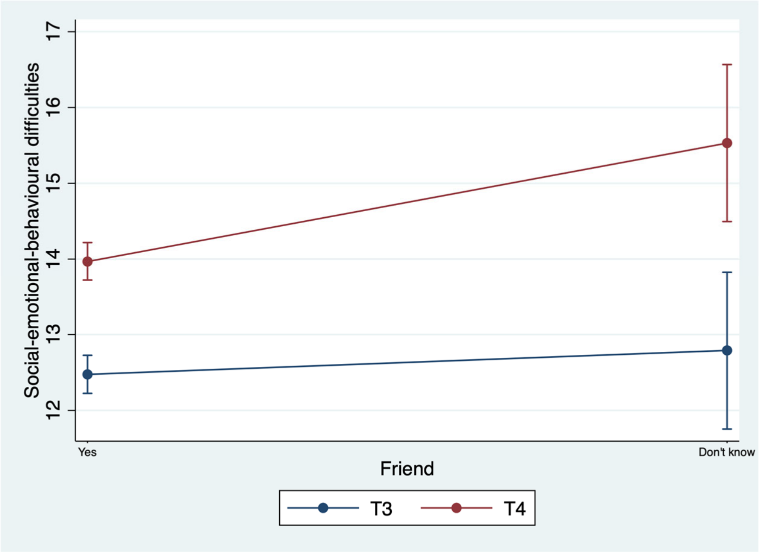

Social-emotional-behavioral difficulties scores (possible range: 0 to 40) are predictive margins (Table 3).

© 2023 Montero-Marin J et al. *JAMA Netw Open.*

eTable 8. 'Student-rated school climate (student level) x home connectedness x time' three-way interaction in the analyses of risk for depression, social-emotional-behavioral difficulties, and well-being (Cohort 2)

	CES-D			
				**LRT**
	**B**	**95% CI**	**p**	**p**
Step 3: *Time*Student-rated school climate*Home connectedness*	-4.39	-6.71, -2.06	**<0.001**	**<0.001**
	**SDQ**			
				**LRT**
	**B**	**95% CI**	**p**	**p**
Step 3: *Time*Student-rated school climate*Home connectedness*	-1.96	-3.12, -0.80	**0.001**	**<0.001**
	**WEMWBS**			
				**LRT**
	**B**	**95% CI**	**p**	**p**
Step 3: *Time*Student-rated school climate*Home connectedness*	1.69	-0.26, 3.65	0.09	**<0.001**

We tested the 'student-rated school climate x home connectedness x time' 3-way interaction. This 3-way interaction was significant in the univariable analyses for risk of depression and social-emotional-behavioral difficulties, but not for well-being. The 3-way interaction term was then included in the previously estimated multivariable models for risk of depression and social-emotional-behavioural difficulties (see eTable 9). LRT: Likelihood-ratio test comparing Model: Student-rated school climate (student level), Home connectedness, Time, *'Student-rated school climate (student level) x Time', 'Home connectedness x Time'* vs Model: Student-rated school climate (student level), Home connectedness, Time, '*Student-rated school climate (student level)' x Time, 'Home connectedness x Time', 'Student-rated school climate (student level) x Home connectedness', 'Student-rated school climate (student level) x* Home *connectedness x Time'*. All models include the design variables, trial-arm status, and the time difference (days) between T3 and T4. CES-D: Center for Epidemiological Studies for Depression Scale (range: 0-60). SDQ: Strengths and Difficulties Questionnaire (Total Difficulties Score; range: 0-40). WEMWBS: Warwick-Edinburgh Mental Well-being Scale (range: 14-70).

© 2023 Montero-Marin J et al. *JAMA Netw Open.*

eTable 9. Multivariable analyses for risk for depression and social-emotional-behavioural difficulties, incorporating the "Time*Student-rated school climate*Home connectedness" three-way interaction (Cohort 2)

	CES-D				SDQ		
	B	95% CI	p		B	95% CI	p
Student characteristics and home environment							
Age	1.24	0.20, 2.28	**0.02**	†		NA	
*Time*Age*	-0.26	-1.13, 0.62	0.57			NA	
Gender female (vs male)	4.78	3.72, 5.84	**<0.001**		1.25	0.69, 1.81	**<0.001**
Gender Other/Prefer not to say (vs male)	5.56	2.47, 8.66	**<0.001**		1.24	-0.46, 2.94	0.15
*Time*Female*	1.58	0.48, 2.67	**0.005**		1.62	1.09, 2.16	**<0.001**
Ethnicity white (vs other ethnic groups)		NA			1.21	0.71, 1.71	**<0.001**
Year group 10 (vs Year group 9)	-1.33	-2.60, -0.07	**0.04**	†	0.20	-0.30, 0.70	0.44
*Time*Year group 10*		NA			-0.25	-0.72, 0.23	0.31
Student-rated school climate (student level)	-4.66	-6.70, -2.63	**<0.001**		-1.96	-3.06, -0.87	**<0.001**
*Time*Student-rated school climate*	5.67	3.44, 7.91	**<0.001**		2.41	1.34, 3.48	**<0.001**
Low risk for mental health difficulties (vs high)	-5.95	-7.09, -4.82	**<0.001**		-3.74	-4.33, -3.15	**<0.001**
*Time*Low risk for mental health difficulties*	2.00	0.75, 3.25	**0.002**		0.96	0.39, 1.54	**0.001**
Household assets	-1.51	-3.85, 0.84	0.21		-0.55	-1.65, 0.55	0.33
*Time*Household assets*	2.51	-0.003, 5.02	**0.05**	†		NA	
Studying conditions	-1.57	-4.25, 1.11	0.26		-1.33	-2.55, -0.10	**0.03** †
*Time*Studying conditions*	-1.08	-4.00, 1.85	0.47			NA	
Home connectedness	-12.06	-14.34, -9.79	**<0.001**		-5.95	-7.12, -4.78	**<0.001**
*Time*Home connectedness*	-9.39	-11.85, -6.93	**<0.001**		-3.80	-4.87, -2.73	**<0.001**
*Student-rated sch climate*Home connectedness*	0.26	-2.32, 2.85	0.84		-1.06	-2.43, 0.31	0.13
*Time*Student-rated sch climate*Home connectedness*	-4.06	-6.90, -1.21	**0.005**		-1.16	-2.50, 0.19	0.09
Home conflicts sometimes (vs lots of times)	0.07	-1.18, 1.33	0.91		0.38	-0.30, 1.06	0.28
Home conflicts rarely	0.34	-1.07, 1.75	0.63		-0.26	-1.00, 0.48	0.49
Home conflicts no/don't know	-0.80	-2.16, 0.57	0.25		-0.78	-1.49, -0.06	**0.03**
*Time*Home conflicts rarely*	-0.16	-1.51, 1.19	0.81		0.22	-0.40, 0.83	0.49
*Time*Home conflicts no/don't know*	-0.91	-2.18, 0.36	0.16		-0.13	-0.71, 0.45	0.66
Friendships							
Friend, don't know (vs yes)	2.20	0.13, 4.26	**0.04**	†	0.28	-0.78, 1.34	0.60
Friend, no (vs yes)	3.06	1.53, 4.60	**<0.001**		2.02	1.18, 2.86	**<0.001**
Friend, prefer not to say (vs yes)	1.96	-1.33, 5.24	0.24		2.08	0.36, 3.80	**0.02** †
*Time*Friend, don't know*	2.76	0.50, 5.01	**0.02**		1.21	0.18, 2.25	**0.02** †
Operational features of the school							
School size	0.00001	-0.003, 0.003	0.99			NA	
*Time*School size*	-0.0001	-0.002, 0.002	0.90			NA	
Coeducational (vs female-only)	1.13	0.08, 2.17	**0.03**	†	0.14	-0.53, 0.80	0.69
*Time*Coeducational*		NA			0.17	-0.42, 0.76	0.57
School quality, req. Improv.	0.01	-2.71, 2.74	0.99			NA	
*Time*School quality, req. improv.*	-2.36	-5.27, 0.55	0.11			NA	
SEL quality rating		NA			-0.09	-0.23, 0.05	0.21
School (during lockdown) some of the time (vs at home)	-0.60	-2.29, 1.10	0.49		0.58	-0.32, 1.47	0.21
Student-rated school climate (sch. Level)	-5.58	-8.35, -2.82	**<0.001**		-1.29	-2.80, 0.23	0.10
Characteristics of the school community							
% SEND support		NA			0.03	-0.03, 0.08	0.37
Students age (sch. Level)	-0.37	-2.59, 1.86	0.75			NA	
*Time*Students age*	2.23	0.29, 4.16	**0.02**	†		NA	

Multivariable analyses, using multilevel linear regressions via Maximum Likelihood estimation and three-level mixed effects models for the analysis of the unique associations between student- and school-level characteristics and changes in adolescents' mental health and well-being between T3 and T4 in Cohort 2 (pandemic), entering those factors that provided significant p-values ($p < 0.05$) in the previous univariable analyses (eTable 5), and including the "Time*Student-rated school climate*Home connectedness" three-way interaction. The continuous student-level factors (age, school climate) were group mean (school-level) centred, and therefore the regression coefficients represent an estimate of the differences in individual effects within schools. The continuous school-level factors were introduced as group means, so that these regression coefficients represent school-level effects (i.e., differences between schools).[29] Regression coefficients of the interaction terms reflect changes relative to the first assessment (i.e., T4 vs. T3). All models controlled for design variables, trial arm allocation, and the time difference (days) between T3 and T4. The 'other ethnic groups' category includes Arab, Asian, Black/African/Caribbean, mixed/multiple ethnic groups, other ethnic groups. CES-D: Center for Epidemiologic Studies for Depression Scale (range: 0-60). SDQ: Strengths and Difficulties Questionnaire (Total Difficulties Score; range: 0-40). † This relationship was no longer significant when the correction for multiple testing was applied. NA: Not applicable given the absence of significant results in the univariable analysis. Those variables that did not show significant univariable associations in any of the outcome variables (i.e., CES-D and SDQ) are omitted. The broader context was not included because the corresponding variables were not applicable.

© 2023 Montero-Marin J et al. *JAMA Netw Open.*

eFigure 10. Relationship between student-rated school climate and risk for depression as a function of home connectedness

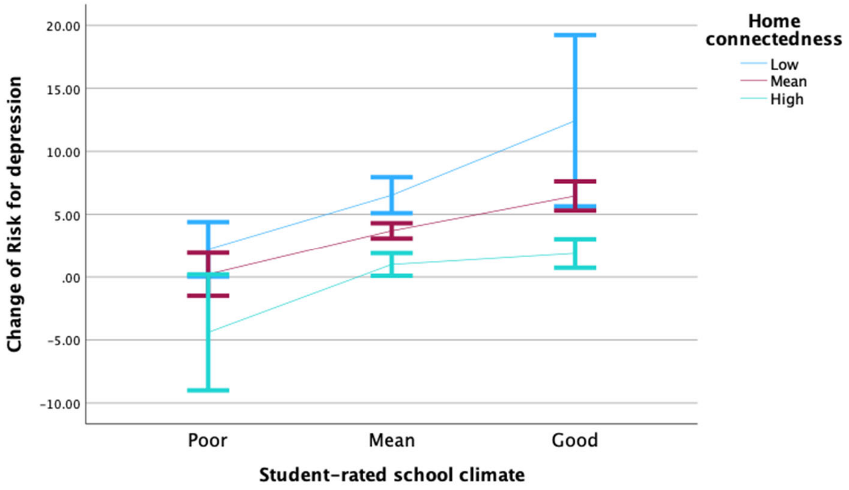

To facilitate interpretation, we modeled the student-rated school climate and family connectedness data using three categories (M±1SD).[30]

© 2023 Montero-Marin J et al. *JAMA Netw Open.*

eTable 10. Univariable and multivariable analyses of factors associated with adolescents' adjustment to lockdown (Cohort 2)

	univariable			multivariable		
	B	95% CI	p	B	95% CI	p
Student characteristics						
Age	-0.04	-0.12, 0.05	0.41		na	
Gender						
female (vs male)	-0.19	-0.31, -0.08	**0.001**	-0.08	-0.20, 0.05	0.24
Other/Prefer not to say (vs male)	-0.30	-0.69, 0.09	0.13		na	
Ethnicity white (vs other ethnic groups)	-0.04	-0.16, 0.07	0.46		na	
Year group 10 (vs Year group 9)	-0.06	-0.17, 0.04	0.24		na	
Student-rated school climate (student level)	0.003	-0.07, 0.07	0.94		na	
Low risk for mental health difficulties (vs high)	0.13	0.02, 0.25	**0.03**	-0.01	-0.15, 0.13	0.88
Home environment						
Household assets	-0.07	-0.31, 0.17	0.59		na	
Studying conditions	0.60	0.32, 0.88	**<0.001**	0.12	-0.20, 0.05	0.47
Home connectedness	0.83	0.64, 1.02	**<0.001**	0.76	0.50, 1.02	**<0.001**
Home conflicts						
sometimes (vs lots of times)	0.15	-0.01, 0.31	0.07		na	
rarely	0.20	0.05, 0.35	**0.009**	-0.05	-0.20, 0.10	0.49
no/don't know	0.36	0.22, 0.50	**<0.001**	0.03	-0.11, 0.17	0.65
Friendships						
Friend						
don't know (vs yes)	-0.29	-0.49, -0.10	**0.003**	-0.18	-0.43, 0.07	0.15
no (vs yes)	-0.08	-0.27, 0.12	0.44		na	
prefer not to say (vs yes)	-0.08	-0.43, 0.28	0.67		na	
Operational features of the school						
School size	0.00	0.00, 0.00	0.78		na	
Student to teacher ratio	-0.02	-0.05, 0.01	0.18		na	
Coeducational (vs female-only)	0.13	0.01, 0.24	**0.03**	0.03	-0.11, 0.17	0.71
School quality						
good (vs outstanding)	0.04	-0.12, 0.20	0.63		na	
req. Improv. (vs outstanding)	-0.05	-0.39, 0.28	0.77		na	
SEL quality rating	-0.01	-0.04, 0.02	0.44		na	
SEL ethos	0.002	-0.01, 0.01	0.60		na	
School attainment	-0.003	-0.01, 0.001	0.14		na	
School time (during lockdown)						
some of the time (vs at home)	-0.15	-0.34, 0.05	0.14		na	
full time	-0.06	-0.24, 0.11	0.47		na	
Teacher-rated school climate (sch. Level)	0.02	-0.17, 0.20	0.86		na	
Student-rated school climate (sch. Level)	-0.23	-0.53, 0.08	0.15		na	
Characteristics of the school community						
% Free school meals (school deprivation)	0.004	-0.01, 0.01	0.33		na	
% SEND support	0.01	-0.01, 0.02	0.26		na	
Students age (sch. Level)	-0.08	-0.29, 0.13	0.44		na	
% Students white	0.001	-0.001, 0.003	0.44		na	
Broader context						
Urbanicity rural (vs urban)	0.04	-0.12, 0.20	0.65		na	
Area level deprivation IMD	0.001	-0.02, 0.02	0.96		na	

Analyses used multilevel linear regressions via Maximum Likelihood estimation and two-level mixed effects models for the analysis of the unique (multivariable) associations between student- and school-level characteristics and adjustment to lockdown (T4) in Cohort 2 (pandemic), entering those factors that provided significant p-values ($p < 0.05$) in the univariable analyses. The continuous student-level factors (age, school climate) were group mean (school-level) centred, and therefore the regression coefficients represent an estimate of the differences in individual effects within schools. The continuous school-level factors were introduced as group means, so that these regression coefficients represent school-level effects (i.e., differences between schools).[29] The 'other ethnic groups' category includes Arab, Asian, Black/African/Caribbean, mixed/multiple ethnic groups, other ethnic groups. The adjustment to lockdown (dependent variable) was measured using a Liket scale from 1 ("life was worse") to 5 ("life was better"). All models controlled for design variables, trial arm allocation, and the time difference (days) between T3-T4. Na: not applicable given the absence of significant results in the univariable analysis. Home connectedness remained significant when we corrected for multiple testing.

© 2023 Montero-Marin J et al. *JAMA Netw Open.*

eTable 11. Univariable and multivariable analyses of factors associated with adolescents' adjustment to return to school (Cohort 2)

	univariable			multivariable		
	**B**	**95% CI**	**p**	**B**	**95% CI**	**p**
Student characteristics						
Age	0.04	-0.04, 0.12	0.32		na	
Gender						
female (vs male)	-0.33	-0.43, -0.22	**<0.001**	-0.27	-0.40, -0.16	**<0.001**
Other/Prefer not to say (vs male)	-0.18	-0.54, 0.18	0.33		na	
Ethnicity white (vs other ethnic groups)	-0.05	-0.17, 0.07	0.42		na	
Year group 10 (vs Year group 9)	-0.06	-0.16, 0.05	0.29		na	
Student-rated school climate (student level)	0.27	0.20, 0.33	**<0.001**	0.19	0.12 0.27	**<0.001**
Low risk for mental health difficulties (vs high)	0.26	0.15, 0.37	**<0.001**	0.14	0.01, 0.27	**0.03** †
Home environment						
Household assets	-0.04	-0.28, 0.19	0.71		na	
Studying conditions	0.74	0.48, 1.00	**<0.001**	0.18	-0.12, 0.48	0.24
Home connectedness	0.84	0.66, 1.03	**<0.001**	0.56	0.31, 0.81	**<0.001**
Home conflicts						
sometimes (vs lots of times)	0.20	0.05, 0.35	**0.008**	0.04	-0.13, 0.20	0.68
rarely (vs lots of times)	0.29	0.15, 0.44	**<0.001**	0.06	-0.10, 0.23	0.45
no/don't know (vs lots of times)	0.28	0.15, 0.42	**<0.001**	-0.05	-0.21, 0.11	0.54
Friendships						
Friend						
don't know (vs yes)	-0.28	-0.46, -0.10	**0.002**	-0.26	-0.49, -0.03	**0.03** †
no (vs yes)	-0.34	-0.52, 0.15	**<0.001**	-0.21	-0.42, 0.004	0.06
prefer not to say (vs yes)	-0.09	-0.42, 0.23	0.57		na	
Operational features of the school						
School size	-0.0002	-0.001, 0.0002	0.32		na	
Student to teacher ratio	0.01	-0.04, 0.06	0.65		na	
Coeducational (vs female-only)	0.14	-0.02, 0.30	0.10		na	
School quality						
good (vs outstanding)	0.02	-0.20, 0.25	0.84		na	
req. Improv. (vs outstanding)	-0.08	-0.50, 0.35	0.73		na	
SEL quality rating	0.02	-0.02, 0.06	0.32		na	
SEL ethos	0.01	-0.01, 0.01	0.32		na	
School attainment	-0.0002	-0.01, 0.01	0.93		na	
School time (during lockdown)						
some of the time (vs at home)	0.23	0.05, 0.41	**0.01**	0.42	0.21, 0.64	**<0.001**
full time (vs at home)	0.26	0.10, 0.43	**0.002**	0.27	0.03, 0.51	**0.03** †
Teacher-rated school climate (school level)	0.11	-0.14, 0.35	0.39		na	
Student-rated school climate (school level)	0.47	0.08, 0.87	**0.02**	0.41	0.06, 0.77	**0.02** †
Characteristics of the school community						
% Free school meals (school deprivation)	-0.003	-0.01, 0.01	0.61		na	
% SEND support	0.004	-0.01, 0.02	0.61		na	
Students age (sch. Level)	-0.19	-0.47, 0.08	0.17		na	
% Students white	-0.0002	-0.003, 0.003	0.92		na	
Broader context						
Urbanicity rural (vs urban)	-0.04	-0.25, 0.17	0.70		na	
Area level deprivation IMD	-0.01	-0.03, 0.02	0.71			

We used multilevel linear regressions via Maximum Likelihood estimation and two-level mixed effects models for the analysis of the unique (multivariable) associations between student- and school-level characteristics and adjustment to lockdown (T4) in Cohort 2 (pandemic), entering those factors that provided significant p-values ($p < 0.05$) in the univariable analyses. The continuous student-level factors (age, school climate) were group mean (school-level) centred, and therefore the regression coefficients represent an estimate of the differences in individual effects within schools. The continuous school-level factors were introduced as group means, so that these regression coefficients represent school-level effects (i.e., differences between schools).[29] The 'other ethnic groups' category includes Arab, Asian, Black/African/Caribbean, mixed/multiple ethnic groups, other ethnic groups. The adjustment to return to school (dependent variable) was measured using a Liket scale from 1 ("life was worse") to 5 ("life was better"). All models controlled for the design variables, trial arm allocation, and the time difference (days) between T3-T4. Na: not applicable because the absence of significant results in the univariable analysis. Home connectedness remained significant when we controlled for multiple testing.

© 2023 Montero-Marin J et al. *JAMA Netw Open.*

eFigure 11. Evidence map of the associations found in the present study

	CES-D	SDQ	WEMWBS	Lockdown	Return
Age					
Time*Age					
Gender female (vs. Male)	***	***	***		***
Gender other/prefer not to say (vs. Male)	***		***		
Time*Gender female	*	***	*		
Time*Gender other/prefer not to say (vs. Male)					
Ethnicity white (vs. Other ethnic groups)		***			
Time*Ethnicity white (vs. Other ethnic groups)					
Year group 10 (vs. Year group 9)					
Time*Year group 10 (vs. Year group 9)					
Student-rated school climate (student level)	***	***	***		***
Time*Student-rated school climate (student level)	***	***	***		
Low risk of mental health problems (vs. high risk)	***	***	***		
Time*Low risk of mental health problems (vs. high risk)	**	**			
Household assets					
Time*Household assets					
Studying conditions					
Time*Studying conditions					
Home connectedness	***	***	***	***	***
Time*Home connectedness	***	***	***		
Student-rated school climate (student-level)*Home connectedness					
Time*Student-rated school climate (student-level)*Home connectedness	*				
Home conflicts sometimes (vs. lots of times)					
Home conflicts rarely (vs. lots of times)					
Home conflicts no/don't know (vs. lots of times)					
Time*Home conflicts sometimes (vs. lots of times)					
Time*Home conflicts rarely (vs. lots of times)					
Time*Home conflicts no/don't know (vs. lots of times)					
Friend don't know (vs. yes)			***		
Friend no (vs. Yes)	***	***	***		
Friend prefer not to say (vs. yes)					
Time*Friend don't know (vs. yes)	*				
Time*Friend no (vs. yes)					
Time*Friend prefer not to say (vs. yes)					
School some of the time (vs. at home)					***
School full time (vs. at home)					
Time*School some of the time (vs. at home)					
Time*School full time (vs. at home)					
School size					
Time*School size					
Student to teacher ratio					
Time*Student to teacher ratio					
Coeducational (vs female-only)					
Time*Coeducational (vs. female-only)					
School quality good (vs. outstanding)					
School quality requires improvement (vs. outstanding)					
Time*School quality good (vs. outstanding)					
Time*School quality requires improvement (vs. outstanding)					
SEL quality rating					
Time*SEL quality rating					
SEL ethos					
Time*SEL ethos					
School attainment					
Time*School attainment					
Teacher-rated school climate (school level)					
Time*Teacher-rated school climate (school level)					
Student-rated school climate (school level)	***				
Time*Student-rated school climate (school level)					
% Free school meals (school deprivation)					
Time*% Free school meals					
% SEND support					
Time*% SEND support					
Students age (school level)					
Time*Students age (school level)					
% Students white					
Time*% Students white					
Urbanicity rural (vs. Urban)					
Time*Urbanicity rural (vs. urban)					
Area level deprivation (IMD)					
Time*Area level deprivation (IMD)					

CES-D: Center for Epidemiological Studies for Depression Scale. SDQ: Strengths and Difficulties Questionnaire (Total Difficulties Score). WEMWBS: Warwick-Edinburgh Mental Well-being Scale. The 'other ethnic groups' category includes Arab, Asian, Black/African/Caribbean, mixed/multiple ethnic groups, other ethnic groups.Lockdown: adjustment to lockdown. Return: adjustment to school return. Models for 'Lockdown' and 'School' do not include the time interaction, as these outcomes were only measured at T4. Cells printed in white represent factors that are not included in the multivariable analyses. Cells printed in red show the varying p-values (the stronger the colour, the lower the p-value). ***p<0.001, **p<0.01, *p<0.05, after correcting for multiple comparisons.

eReferences

[1]Timeline of UK government coronavirus lockdowns and measures, March 2020 to December 2021. https://www.instituteforgovernment.org.uk/sites/default/files/timeline-coronavirus-lockdown-december-2021.pdf. Accessed June 30, 2022.

[2]Radloff LS. The use of the center for epidemiologic studies depression scale in adolescents and young adults. *J Youth Adolesc*. 1991;20(2):149-166.

[3]Goodman R. Psychometric properties of the Strengths and Difficulties Questionnaire. *J Am Acad Child Adolesc Psychiatry*. 2001;40:1337-1345.

[4]Tennant R, Hiller L, Fishwick R, et al. The Warwick-Edinburgh mental well- being scale (WEMWBS): development and UK validation. *Health Qual Life Outcomes*. 2007;5:63.

[5]Clarke A, Friede T, Putz R, et al. Warwick-Edinburgh Mental Well-being Scale (WEMWBS): validated for teenage school students in England and Scotland. A mixed methods assessment. *BMC Public Health*. 2011;11:487.

[6]Ford T, Degli Esposti M, Crane C, et al. The role of schools in early adolescents' mental health: Findings from the MYRIAD study. *J Am Acad Child Adolesc Psychiatry*. 2021;60(12):1467-1478.

[7]Association of Alaska School Boards. School climate and connectedness survey [measurement instrument]. Juneau: Association of Alaska School Boards; 2015.

[8]Nylund KL, Asparouhov T, Muthén BO. Deciding on the number of classes in latent class analysis and growth mixture modeling: A Monte Carlo simulation study. *Struct Equ Modeling*. 2007;14(4):535-569.

[9]Bakk Z, Vermunt JK. Robustness of stepwise latent class modeling with continuous distal outcomes. *Struct Equ Modeling*. 2016;23(1):20-31.

[10]Montero-Marin J, Allwood M, Ball S, et al. School-based mindfulness training in early adolescence: what works, for whom and how in the MYRIAD trial? *Evid Based Ment Health*. 2022;25(3):117-124.

[11]Hartley JE, Levin K, Currie C. A new version of the HBSC Family Affluence Scale – FAS III: Scottish Qualitative Findings from the International FAS Development Study. *Child Indic Res*. 2016;9:233-245.

[12]Eisenberg, ME, Resnick MD. Suicidality among gay, lesbian and bisexual youth: the role of protective factors. *J Adolesc Health*. 2006;39(5):662-668.

[13]Office for Standards in Education, 2016. https://www.gov.uk/government/organisations/ofsted. Accessed January 30, 2020.

[14]Department of Education. Personal, social, health and economic (PSHE) education: A mapping study of the prevalent models of delivery and their effectiveness. London; 2010. Contract No.: DFE-RR080.

[15]Weare K, Nind M. Mental health promotion and problem prevention in schools: what does the evidence say? *Health Promot Int*. 2011;26 Suppl 1:i29-i69.

[16]Department for Education, 2016. https://assets.publishing.service.gov.uk/government/uploads/system/uploads/attachment_

© 2023 Montero-Marin J et al. *JAMA Netw Open.*

data/file/561021/Progress_8_and_Attainment_8_how_measures_are_calculated.pdf. Accessed January 30, 2020.

[17]Department for Education. *Statistics: performance tables*. 2013. https://www.gov.uk/government/collections/statistics-performance-tables. Accessed January 30, 2020.

[18]Department for Education. *Progress 8: How Progress 8 and Attainment 8 measures are calculated*. 2017. http://dera.ioe.ac.uk/id/eprint/28143. Accessed January 30, 2020.

[19]Index of Multiple Deprivation 2015. National statistics. English indices of deprivation 2015. https://www.gov.uk/government/statistics/english-indices-of-deprivation-2015. Accessed January 30, 2020.

[20] Jakobsen JC, Gluud C, Wetterslev J, Winkel P. When and how should multiple imputation be used for handling missing data in randomised clinical trials - a practical guide with flowcharts. *BMC Med Res Methodol*. 2017;17(1):162.

[21]Kuyken W, Nuthall E, Byford S, et al. The effectiveness and cost-effectiveness of a mindfulness training programme in schools compared with normal school provision (MYRIAD): study protocol for a randomised controlled trial. *Trials*. 2017;18(1):194.

[22]Montero-Marin J, Nuthall E, Byford S, et al. Update to the effectiveness and cost-effectiveness of a mindfulness training programme in schools compared with normal school provision (MYRIAD): study protocol for a randomised controlled trial. *Trials*. 2021;22(1):254.

[23]Kenward M, Roger J. Small sample inference for fixed effects from restricted maximum likelihood. *Biometrics*. 1997;53(3):983-997.

[24]Cohen J, Cohen P, West SG, Aiken LS. Applied Multiple Regression/Correlation Analysis for the Behavioral Sciences. Routledge; 2003.

[25]Kuyken W, Ball S, Crane C, et al. Effectiveness and cost-effectiveness of universal school-based mindfulness training compared with normal school provision in reducing risk of mental health problems and promoting well-being in adolescence: the MYRIAD cluster randomised controlled trial. *Evid Based Ment Health*. 2022;25(3):99-109.

[26]Department of Education. Schools, pupils and their characteristics: January 2020 London, UK2020. https://www.gov.uk/government/statistics/schools-pupils-and-their-characteristics-january-2020. Accessed January 30, 2022.

[27]Briere FN, Pascal S, Dupere V, Janosz M. School environment and adolescent depressive symptoms: A multi-level longitudinal study. *Pediatrics*. 2013;131(3)e702-e708.

[28]British means and standard deviations for the SDQ by age band. https://www.sdqinfo.org/norms/UKNorm3.pdf. Accessed April 26, 2021.

[29]Kreft IG, de Leeuw J, Aiken LS. The effect of different forms of centering in hierarchical linear models. Multivariate Behav Res. 1995;30(1):1-21.

[30] KJ, Curran PJ, Bauer DJ. (2006). Computational tools for probing interactions in multiple linear regression, multilevel modeling, and latent curve analysis. Journal of Educational and Behavioral Statistics, 31,437e448.

© 2023 Montero-Marin J et al. *JAMA Netw Open.*
