# Supplementary material for: Young People’s Mental Health Changes, Risk, and Resilience During the COVID-19 Pandemic
Source: JAMA Netw Open. 2023 Sep 21;6(9):e2335016. doi: 10.1001/jamanetworkopen.2023.35016 (PMC10514742; doi:10.1001/jamanetworkopen.2023.35016)
Supplement: Supplement 2. — Nonauthor Collaborators. The MYRIAD Team [file jamanetwopen-e2335016-s002.pdf]

Supplemental Online Content: Nonauthor Collaborators
*First name, last name, and suffix (if applicable) are required and will appear in PubMed.

*Group Name(s): The MYRIAD Team							
***First Name and Middle Initial(s)**	***Last Name**	***Suffix (eg, Jr, III)**	Academic Degrees	Institution	Location (city, state/province, country)	Role or Contribution, eg, chair, principal investigator	Group (if more than 1 Group listed in the byline) and/or Subgroup (eg, Steering Committee)
Saz	Ahmed		PhD	University College London			
Louise	Aukland		PGCE	University of Oxford			
Susan	Ball		MSc	University of Exeter			
Triona	Casey		MSc	University of Oxford			
Catherine	Crane		PhD	University of Oxford			
Katherine	De Wilde		PGCE	University of Oxford			
Darren	Dunning		PhD	University of Cambridge			
Poushali	Ganguli		MSc	Kings College London			
Ben	Jones		PhD	University of Exeter			
Nils	Kappelmann		MSc	Max Planck Institute of Psychiatry and International Max Planck Research School for Translational Psychiatry			
Maria E.J.	Kempnich		PhD	University of Oxford			
Konstantina	Komninidou		BEd	University of Oxford			
Rachel	Knight		MSc	University of Cambridge			
Suzannah	Laws		BSc	University of Oxford			
Liz	Lord		MSc	University of Oxford			
Emma	Medlicott		MSc	University of Oxford			
Jenna	Parker		MSc	University of East Anglia			
Blanca	Piera Pi-Sunyer		MSc	University College London			
Isobel	Pryor-Nitsch		MSc	University of Oxford			
Jem	Shackleford		MA, MSc	University of Oxford			
Laura	Taylor		PhD	University of Oxford			
Brian	Wainman		BEng	Plymouth University			
Lucy	Warriner		BSc	University of Cambridge			
