# Supplementary material for: Young People’s Mental Health Changes, Risk, and Resilience During the COVID-19 Pandemic
Source: JAMA Netw Open. 2023 Sep 21;6(9):e2335016. doi: 10.1001/jamanetworkopen.2023.35016 (PMC10514742; doi:10.1001/jamanetworkopen.2023.35016)
Supplement: Supplement 3. — Data Sharing Statement [file jamanetwopen-e2335016-s003.pdf]

Data Sharing Statement

Montero-Marin. Young People's Mental Health Changes, Risk, and Resilience During the COVID-19 Pandemic. *JAMA Netw Open*. Published September 21, 2023. doi:10.1001/jamanetworkopen.2023.35016

Data
Data available: Yes
Data types: Deidentified participant data, Data dictionary
How to access data: Information about accessing data is set out on our study website https://myriadproject.org
When available: With publication

Supporting Documents
Document types: None

Additional Information
Who can access the data: Those making an appropriate application as set out on our website.
Types of analyses: Those with a clear research rationale that do not duplicate existing work or work in progress.
Mechanisms of data availability: Data are available on reasonable request. The de-identified baseline data and codebook from the MYRIAD trial are available from Prof Kuyken (willem.kuyken@psych.ox.ac.uk) on request (release of data is subject to an approved proposal and a signed data access agreement).
